# BRD7 suppresses tumor chemosensitivity to CHK1 inhibitors by inhibiting USP1-mediated deubiquitination of CHK1

**DOI:** 10.1038/s41420-023-01611-x

**Published:** 2023-08-25

**Authors:** Lemin Li, Linchen Wang, Dian Liu, Yongchao Zhao

**Affiliations:** 1 Department of Hepatobiliary and Pancreatic Surgery, the First Affiliated Hospital, Zhejiang University School of Medicine, Hangzhou, China; 2 Zhejiang Provincial Key Laboratory of Pancreatic Disease, the First Affiliated Hospital, Zhejiang University School of Medicine, Hangzhou, China; 3 Institute of Translational Medicine, Zhejiang University School of Medicine, Hangzhou, China; 4 Cancer Center, Zhejiang University, Hangzhou, China

**Keywords:** Ubiquitylation, Deubiquitylating enzymes

## Abstract

Checkpoint kinase 1 (CHK1), a key effector in the cellular response to DNA lesions, is a crucial component of all cell cycle checkpoints. Recent reports have revealed that CHK1 is highly expressed in numerous cancer types in the clinical settings. However, the mechanisms underlying the regulation of CHK1 expression in tumor cells remain unclear. Here, we report that CHK1 is negatively regulated by the bromodomain-containing protein 7 (BRD7). Specifically, BRD7 silencing increased CHK1 (but not CHK2) expression at both mRNA and protein levels, in a p53-independent manner in multiple tumor cell lines. Furthermore, BRD7 silencing stabilized CHK1 via reducing its ubiquitination. Mechanistically, BRD7 knockdown not only increased the levels of USP1, a deubiquitinase for CHK1, but also promoted the interaction between CHK1 and USP1, subsequently enhancing the de-ubiquitination of CHK1. USP1 knockdown abrogated BRD7 silencing-induced CHK1 induction. Biologically, the increased expression of CHK1 in tumor cells caused by BRD7 silencing significantly increased cell sensitivity to CHK1 inhibitors by enhancing tumor cell apoptosis, and this effect was reversed by the simultaneous knockdown of CHK1 or USP1. Taken together, our findings suggest that BRD7 is a potential genetic or drug target that may help to improve the efficacy of chemotherapeutic drugs targeting CHK1 in combinatorial therapy.

## Introduction

Checkpoint kinase 1 (CHK1), a core effector of the DNA damage response (DDR), is a Ser/Thr kinase that represents the primary component of all cell cycle checkpoints. Activated CHK1 blocks the cell cycle and allows the cells to repair DNA damage prior to the next stage, or induces cell death if the damage is irreparable [1–3]. In addition, CHK1 is involved in the control of replication origin firing [4], the stabilization of stalled replication forks [5], and the promotion of homologous recombination (HR) [6, 7]. CHK1 dysfunction may lead to abnormal cell cycle progression, involving abnormal DNA repair, cell-cycle regulation, gene transcription, and cell death, resulting in genome instability and oncogenic transformation of cells [1, 3, 8–10]. Several reports have demonstrated the overexpression of CHK1 in multiple cancer types, such as lung cancer [11], nasopharyngeal carcinoma [12], and gastric cancer [8]. CHK1 overexpression significantly increases resistance to chemoradiotherapy in cancer patients [13, 14], suggesting that CHK1 inhibitors may improve the efficacy of tumor chemo-radiosensitization. However, the mechanisms underlying CHK1 dysregulation remain unknown. Therefore, it is important to elucidate the mechanism of CHK1 regulation in tumor cells, which may provide theoretical evidence for effective cancer therapy through modulation of these mechanisms and identification of molecular targets.

The overall activity of CHK1 is regulated by various mechanisms including phosphorylation and ubiquitination. For instance, CHK1 is activated via phosphorylation at residues Ser^345^ and Ser^317^ to regulate multiple signaling pathways, while it is inactivated through dephosphorylation [15, 16] and ubiquitination; K63-linked ubiquitination affects protein localization and activation [17, 18] and K48-linked ubiquitination mediates protein degradation [19–22]. Given the abnormal expression of CHK1 in tumor cells, it is intriguing to explore whether changes in CHK1 expression and activity are related to phosphorylation and ubiquitination, which could provide new insights into chemotherapeutic drugs targeting CHK1 for anti-cancer therapy.

Bromodomain-containing protein 7 (BRD7), a member of the bromodomain-containing protein family, forms a PBAF-specific SWI/SNF chromatin-remodeling complex with other members that plays a role in regulating transcription [23, 24]. Structurally, BRD7 contains an evolutionarily conserved bromodomain (BRD), which acts as a chromatin-targeting module that recognizes acetylated histone tails to positively or negatively regulate the transcription of target genes [25–27]. Accumulating evidence suggests that BRD7 has a role in regulating tumorigenesis. Reduced expression of BRD7 has been reported in various types of tumors, including nasopharyngeal carcinoma, breast cancer, and colorectal carcinoma, indicating that BRD7 functions as a tumor suppressor [28–31]. BRD7 regulates cellular growth, mobility, and apoptosis by interacting with several key proteins such as p53 and BRCA1 [25, 26]. In addition, BRD7 arrests cell cycle progression from G1 to S phase by regulating cell cycle-related molecules, such as cyclin D1 and E2F3, in nasopharyngeal carcinoma [31–33]. Moreover, BRD7 is recruited to the active transcription regions at the damaged chromatin via ATM signaling, which is required for transcriptional repression and DNA repair [34]. These findings indicate the crucial roles of BRD7 in cell cycle control, checkpoint establishment, and DNA damage response and repair. Interestingly, BRD7 has recently been reported to regulate protein stability via ubiquitin-dependent proteasome pathway to inhibit cell growth [35, 36], suggesting its non-transcriptional functions in tumor cells. As CHK1 is a key Ser/Thr kinase involved in the control of cell cycle checkpoints and DNA damage response, and may serve as an ideal therapeutic drug target [3], it would be intriguing to explore the underlying correlation between BRD7 and CHK1 in tumor cells and the role of the BRD7-CHK1 axis in chemotherapy.

In this study, we demonstrated that BRD7 binds to CHK1 and regulates its degradation through ubiquitination by negatively regulating its deubiquitinase USP1. A significant increase in CHK1 levels and activity in tumor cells caused by the silencing of BRD7 enhances tumor cell sensitivity to CHK1 inhibitors, leading to increased cell death via apoptosis, which is reversed by the simultaneous knockdown of CHK1 or USP1. Our study suggests that BRD7 may represent a potential genetic or drug target for combinatorial therapy to improve the efficacy of chemotherapeutic drugs targeting CHK1.

## Results

### BRD7 negatively regulates the expression of CHK1 in tumor cells

**Fig. 1:**
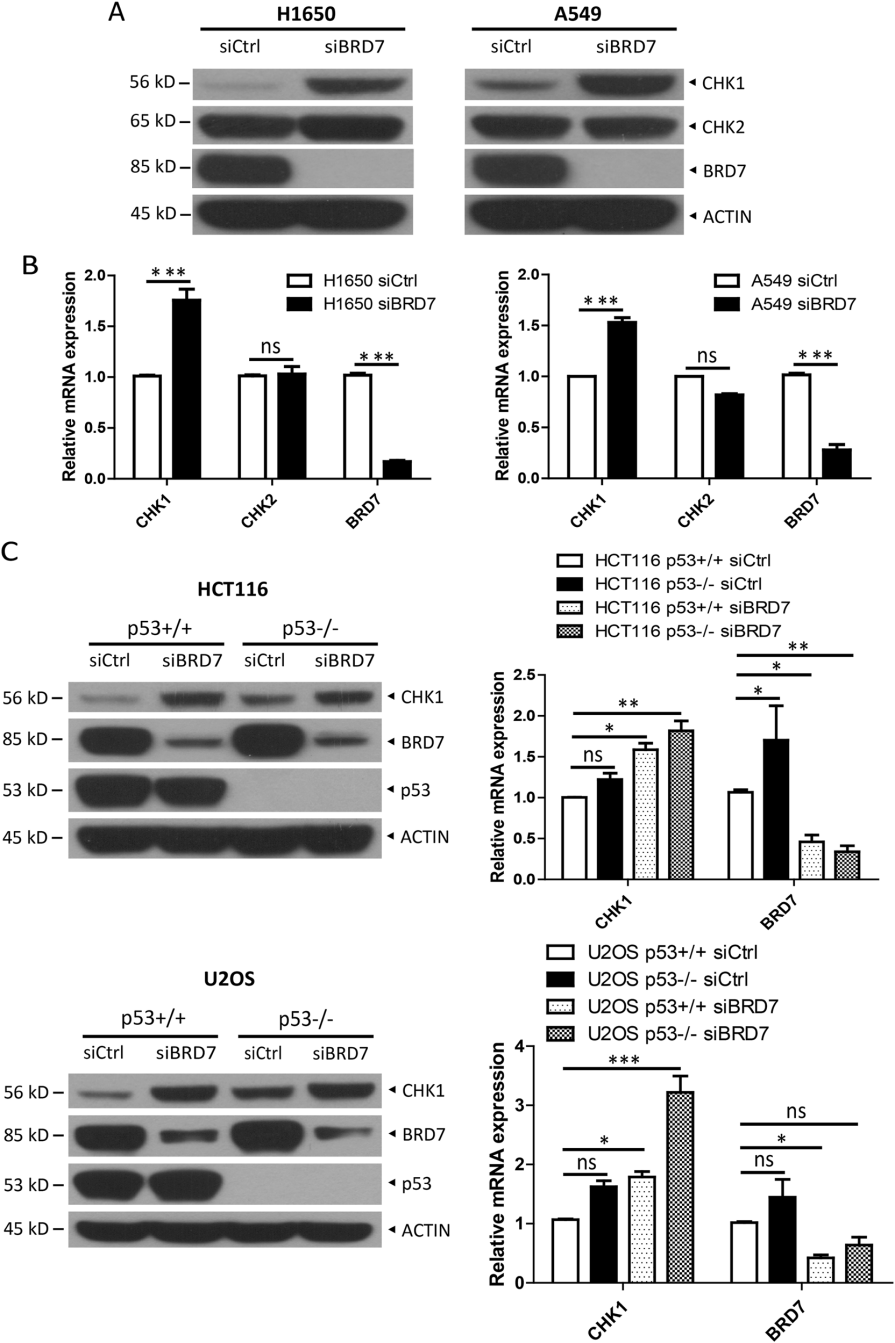
BRD7 negatively regulates CHK1 expression in tumor cells. Silencing of BRD7 increases CHK1 levels but not CHK2. Cells transfected with siRNA targeting BRD7 (siBRD7) or scrambled control siRNA (siCtrl) were harvested at 48 h, followed by immunoblotting (IB) with the indicated antibodies (**A**) or quantitative real-time PCR (RT-qPCR) analysis (**B**). **C** BRD7 regulates the transcription of CHK1 in a p53-independent manner. HCT116 and U2OS cells with or without p53 deletion were transfected with siBRD7 or siCtrl for 48 h, followed by RT-qPCR analysis or IB with the indicated antibodies. Data from three independent experiments were expressed as mean ± SEM, *p < 0.05, **p < 0.01, ***p < 0.001, ns: not significant.

Given that BRD7 is involved in cell cycle progression by regulating crucial cell cycle-associated pathways, such as the Rb/E2F pathway [28], we explored the mechanism by which BRD7 regulates CHK1. We first examined CHK1 protein levels in H1650 and A549 cells after silencing BRD7. As shown in Fig. 1A, silencing of BRD7 significantly increased the protein levels of CHK1 but not of CHK2, and this effect was consistently observed in multiple tumor cell lines, including U2OS, H1792, H358, HCT116, SKBR3, and MCF7 (Fig. S1A), suggesting that BRD7 negatively regulates the expression of CHK1 protein. As BRD7 is well known for its transcriptional regulation, we next detected the changes in the mRNA levels of both CHK1 and CHK2. BRD7 silencing increased the mRNA levels of CHK1, but not of CHK2, compared to the control group, which was consistent with the protein level analysis (Fig. 1B). BRD7 usually serves as a cofactor and coordinates with transcription factors to regulate target gene transcription, which prompted us to search for transcription factors that are involved in the regulation of CHK1 by BRD7. We first examined p53, which serves as a transcriptional suppressor of CHK1 [8]. We found that BRD7 knockdown increased the mRNA and protein levels of CHK1 in HCT116 and U2OS cells, regardless of p53 status, suggesting that the regulation of BRD7 on CHK1 was independent of p53 (Fig. 1C). Furthermore, we detected the alternation of two other transcriptional activators involved in CHK1 transcription, E2F1 [8] and E4F1 [37], and found that, upon BRD7 knockdown, their levels altered in a cell context-dependent manner (Fig. S1A, B). In addition, E2F1 or E4F1 knockdown increased the levels of CHK1 (Fig. S1C), which is inconsistent with the results of previous studies [8, 37], suggesting that their roles in transcriptional regulation are cell or context-dependent. Thus, the transcription factor (s) that mediate CHK1 transcription coordinated by BRD7 need to be investigated further. Taken together, our results demonstrate that the expression of CHK1 is negatively regulated by BRD7.

### BRD7 binds to CHK1 and negatively regulates CHK1 stability through ubiquitination

**Fig. 2:**
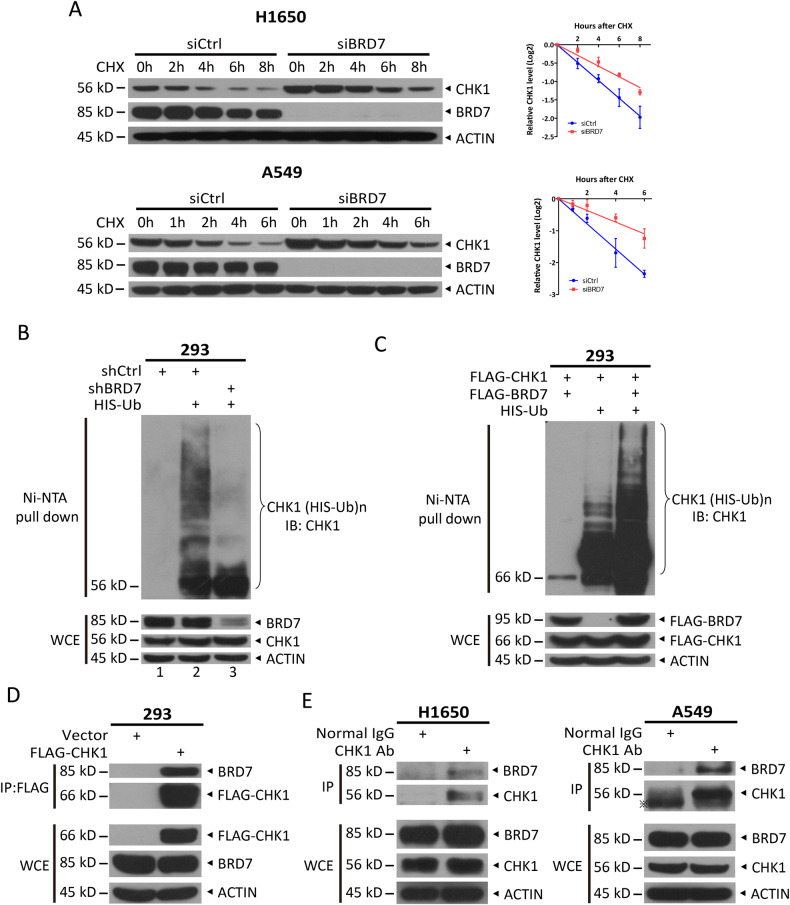
BRD7 binds to CHK1 and negatively regulates CHK1 stability through ubiquitination. **A** Silencing of BRD7 extends the half-life of CHK1 protein. Cells transfected with the indicated siRNAs were treated with 100 µg/mL CHX for the indicated times and then subjected to immunoblotting (IB) with the indicated antibodies. Densitometry quantification was performed with ImageJ, and the decay curves are shown (right), The decay curve is calculated by dividing the densitometry quantification after normalized with the corresponding ACTIN at the indicated time points and that of the control (0 h), expressed as the log with base 2. Data from three independent experiments were expressed as mean ± SEM. BRD7 promotes CHK1 polyubiquitination. HEK293 cells (**C**) or HEK293 cells infected with a lentivirus expressing indicated shRNAs (**B**) were transfected with the indicated plasmids for 48 h and then treated with 20 µM MG132 for 5 h, followed by Ni-bead pull-down and IB with the indicated antibodies. **D** Ectopically expressed CHK1 binds to endogenous BRD7. HEK293 cells transfected with the indicated plasmids were treated with 20 µM MG132 for 5 h and then harvested for IP with anti-FLAG beads. Subsequently, IB was performed with the indicated antibodies. **E** CHK1 binds to endogenous BRD7. A549 and H1650 cells were lysed for IP with CHK1 antibody or normal IgG, followed by IB for BRD7. Whole-cell lysates (WCE) were subjected to IB with the indicated antibodies.

The protein level of CHK1 depends on the amount of mRNA and the stability of the protein. MG132, a proteasome inhibitor, partially blocked the reduction in exogenous CHK1 induced by BRD7 overexpression (Fig. S2), suggesting that BRD7 could regulate CHK1 degradation. Next, we determined whether BRD7 regulates the protein stability of CHK1 by shortening its half-life. Silencing of BRD7 significantly extended the half-life of CHK1 (Fig. 2A). As most of the intracellular proteins are degraded by the ubiquitin-proteasome system, we next examined whether manipulation of BRD7 levels alters CHK1 ubiquitination. Indeed, the in vivo ubiquitination assay showed that the shRNA-based knockdown of BRD7 dramatically blocked the ubiquitination of endogenous CHK1 (Fig. 2B, lanes 3 versus 2), whereas BRD7 overexpression promoted the ubiquitination of exogenously expressed CHK1 (Fig. 2C). Furthermore, BRD7 was readily detected in immunoprecipitants using ectopically expressed FLAG-CHK1, indicating an interaction between BRD7 and CHK1 (Fig. 2D). This interaction was verified by the finding that endogenous CHK1 could pull down endogenous BRD7 under physiological growth conditions (Fig. 2E). Taken together, our results demonstrate that BRD7 binds to CHK1 and negatively regulates CHK1 stability by promoting its ubiquitination.

### BRD7 regulates CHK1 stability and ubiquitination in a USP1-dependent manner

**Fig. 3:**
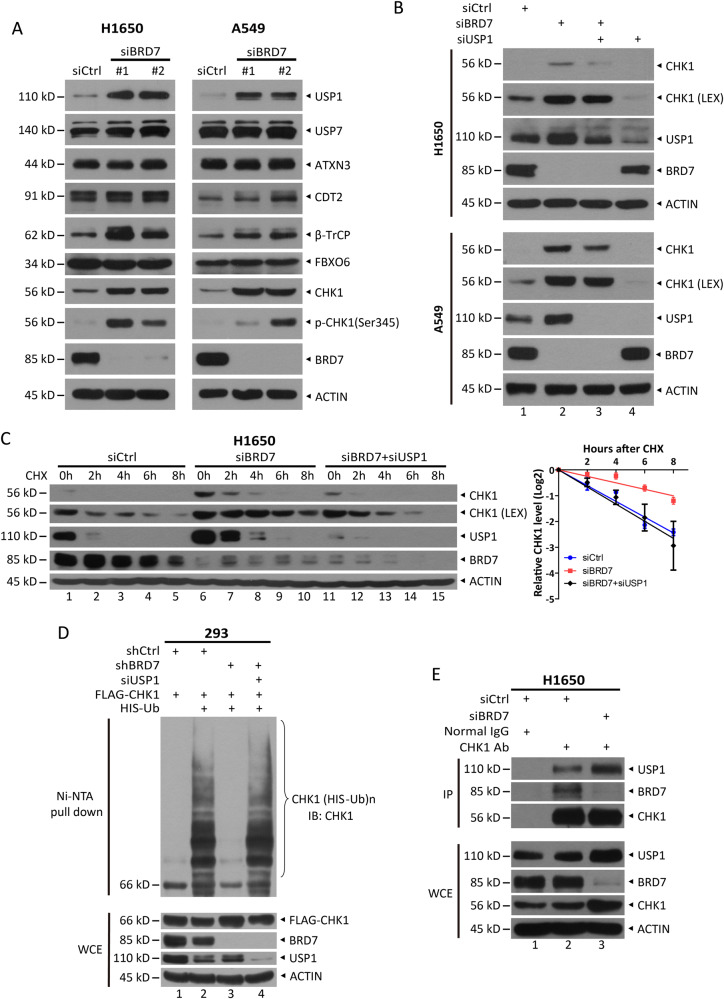
BRD7 regulates CHK1 stability and ubiquitination in a USP1-dependent manner. **A** Silencing of BRD7 increases USP1 levels. Cells transfected with siRNA targeting BRD7 or scrambled control siRNA were harvested for immunoblotting (IB) with the indicated antibodies. USP1 silencing reverses the effect of BRD7 on CHK1. Cells transfected with the indicated siRNAs were harvested for IB with the indicated antibodies (**B**), or treated with 100 µg/mL CHX for the indicated time periods and then subjected to IB with the indicated antibodies (**C**). Densitometry quantification was performed using ImageJ, and the decay curve is shown (right). Data from three independent experiments were expressed as mean ± SEM. **D** USP1 silencing reverses the effect of BRD7 on CHK1 polyubiquitination. HEK293 cells infected with lentivirus expressing the indicated shRNAs were transfected with the indicated plasmids or siRNAs for 48 h and then treated with 20 µM MG132 for 5 h, followed by Ni-bead pull-down assay and IB with the indicated antibodies. **E** Silencing of BRD7 enhances the interaction between USP1 and CHK1. H1650 cells transfected with siRNA targeting BRD7 or scrambled control siRNA were lysed for IP with CHK1 antibody or normal IgG, followed by IB with the indicated antibodies. Whole-cell lysates (WCE) were subjected to IB with the indicated antibodies. LEX: longer exposure.

Given that BRD7 is not a ubiquitin ligase, to further reveal the mechanism underlying BRD7-promoted CHK1 ubiquitination, we examined E3 ligases targeting CHK1 for degradation, including CRL4^CDT2^, SCF^β-TrCP^, and SCF^FBXO6^ [19–21], and deubiquitinases that stabilize CHK1, including USP1, USP7, and ATXN3 [38–40]. Interestingly, we found that BRD7 knockdown had no effect on, but even increased the levels of E3 ligases, suggesting that BRD7 may not regulate CHK1 ubiquitination by modulating E3 ligases (Figs. 3A and S3A). Moreover, BRD7 knockdown specifically increased USP1 levels but had no effect on the levels of USP7 and ATXN3, suggesting that increased USP1 may deubiquitinate and stabilize CHK1 (Figs. 3A and S3A). Given that USP1 positively regulates CHK1 stabilization (Fig. S3B), to further examine whether the regulation of BRD7 on CHK1 is dependent on USP1, we performed a rescue experiment and found that USP1 silencing reversed the increase in CHK1 level (Fig. 3B, lanes 3 versus 2 and 1; Fig. 3C, lanes 11 versus 6 and 1) and protein half-life of CHK1 (Fig. 3C, lanes 11–15 versus 6–10 and 1–5) caused by BRD7 knockdown. Moreover, the in vivo ubiquitination assay showed that USP1 silencing reversed the effect of BRD7 on CHK1 polyubiquitination (Fig. 3D, lanes 4 versus 3 and 2). Interestingly, USP1 silencing only partially reversed the protein levels of CHK1 (Fig. 3B, lanes 3 versus 2 and 1) and totally reversed the protein half-life and ubiquitination of CHK1, which further indicates BRD7 could negatively regulate CHK1 transcription. Finally, more endogenous USP1 was detected in immunoprecipitants with CHK1 upon BRD7 knockdown (Figs. 3E and S3C, lanes 3 versus 2), indicating that BRD7 knockdown facilitates USP1 binding to CHK1, leading to CHK1 deubiquitination. Taken together, these results demonstrate that the regulation of CHK1 stability and ubiquitination by BRD7 is dependent on the deubiquitinase USP1.

### BRD7 deficiency promotes DNA replication, cell proliferation, and survival in a CHK1-dependent manner

**Fig. 4:**
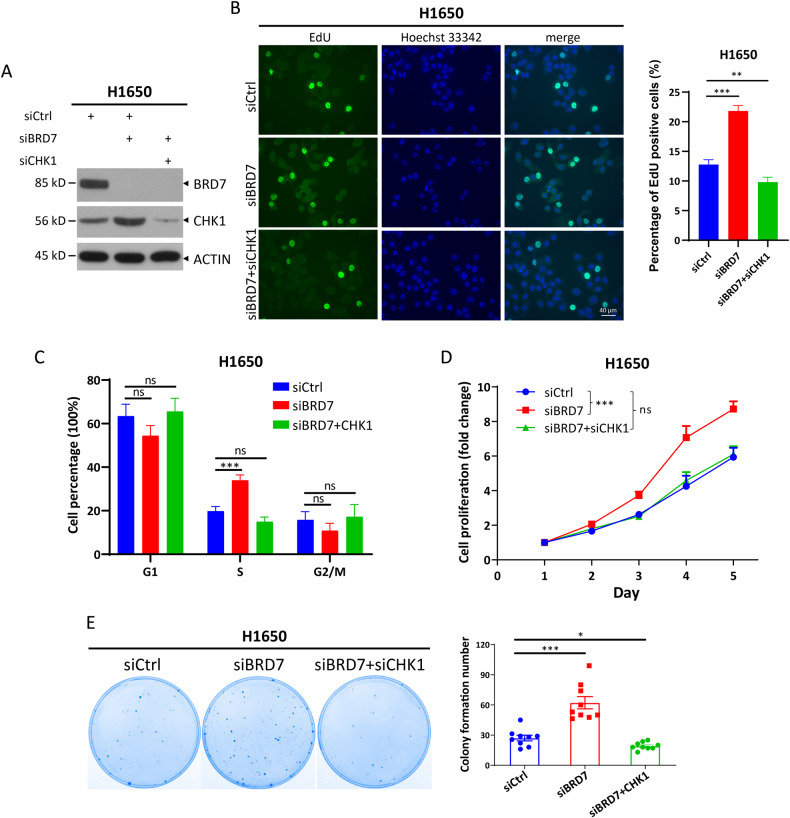
BRD7 deficiency promotes DNA replication, cell proliferation, and survival in a CHK1-dependent manner. **A** Silencing of BRD7 increases CHK1 levels and simultaneous silencing of BRD7 and CHK1 downregulates CHK1 levels. H1650 cells transfected with indicated siRNA were harvested at 48 h, followed by immunoblotting (IB) with the indicated antibodies. **B** Silencing of BRD7 increases DNA replication, while simultaneous silencing of BRD7 and CHK1 suppresses DNA replication. H1650 cells transfected with indicated siRNA were performed EdU incorporation assay using BeyoClick™ EdU Cell Proliferation Kit. The percentage of EdU-positive cells in the total number of cells was determined from at least five random fields. Silencing BRD7 increases the percentage of S phase of cell cycle (**C**), cell proliferation (**D**) and cell survival (**E**) in a CHK1-dependent manner. H1650 cells were transfected with the indicated siRNA oligos for 48 h and then split for flow cytometry (**C**), CCK8 assay (**D**), and clonogenic survival assay (**E**). Data from three independent experiments are expressed as mean ± SEM, *p < 0.05, **p < 0.01, ***p < 0.001; ns: not significant.

Next, we examined the biological consequences of CHK1 up-regulation induced by BRD7 deficiency in tumor cells. Previous study showed that CHK1 plays key roles in controlling replication initiation and replication fork progression [41]. First, we investigated whether the induction of CHK1 caused by BRD7 deficiency regulates DNA replication, and found that BRD7 knockdown significantly promoted the incorporation of 5-ethynyl-2′-deoxyuridine (EdU), a marker indicating DNA replication, and simultaneous CHK1 knockdown reversed the increased EdU incorporation in H1650 and A549 cells (Figs. 4A, B and S4A, B). Consistently, flow cytometric analysis showed that simultaneous CHK1 knockdown rescued the increased percentage of S-phase caused by BRD7 knockdown (Figs. 4C and S4C). Moreover, BRD7 knockdown obviously promoted cell proliferation and survival, while silencing of both BRD7 and CHK1 abrogated the induction of cell proliferation and survival (Figs. 4D, E and S4D, E). Taken together, these results demonstrate that BRD7 deficiency promotes DNA replication, cell proliferation, and survival in a CHK1-dependent manner.

### BRD7 deficiency sensitizes tumor cells to CHK1 inhibitors by promoting apoptosis via accumulation of CHK1

**Fig. 5:**
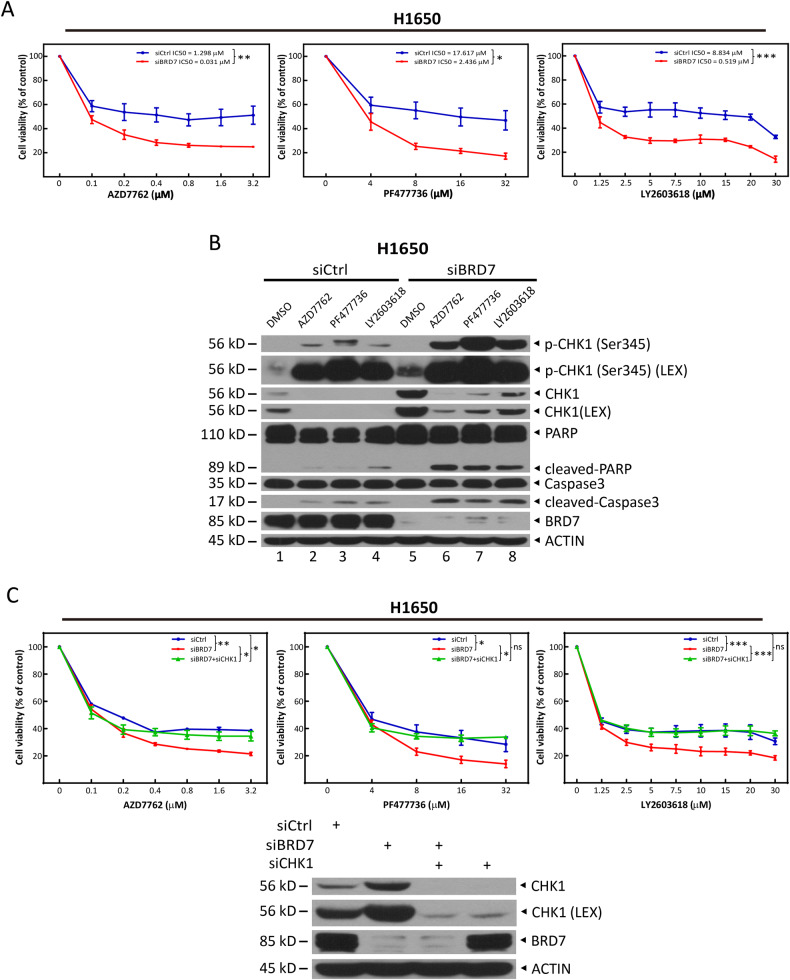
BRD7 deficiency sensitizes tumor cells to CHK1 inhibitors via accumulation of CHK1. **A** Silencing of BRD7 sensitizes tumor cells to CHK1 inhibitors. H1650 cells transfected with siRNA targeting BRD7 or scrambled control siRNA were plated in triplicate in 96-well plates and treated with various concentrations of AZD7762, PF477736, and LY2603618 for 72 h for the CCK8 assay. Data from three independent experiments are expressed as mean ± SEM, *p < 0.05, **p < 0.01, ***p < 0.001. **B** Silencing of BRD7 promotes CHK1 inhibitor-induced apoptosis. H1650 cells transfected with siRNA targeting BRD7 or scrambled control siRNA were treated with AZD7762, PF477736, or LY2603618 for 24 h and then subjected to immunoblotting (IB) with the indicated antibodies. **C** Simultaneous CHK1 knockdown reverses the BRD7 deficiency-induced chemosensitization to CHK1 inhibitors. Cells transfected with the indicated siRNAs were plated in triplicate in 96-well plates, treated with various concentrations of the three CHK1 inhibitors for 72 h, and then subjected to the CCK8 assay. The cells remaining in the plates were subjected to IB with the indicated antibodies (bottom). Data from three independent experiments are expressed as mean ± SEM; *p < 0.05, **p < 0.01, ***p < 0.001; ns: not significant. Given that BRD7 knockdown significantly increased cell proliferation in H1650 cells, to maintain the equivalent number of cells in each group in response to drug treatment, 1 × 10^3^ or 1.2 × 10^3^ cells per well were used for siRBD7 group and 3 × 10^3^ cells per well for other groups. LEX: longer exposure.

It is well known that the level of CHK1 positively correlates the sensitization of tumor cells to CHK1 inhibitors [42, 43]. Indeed, the tumor cells were significantly sensitized to the three CHK1 inhibitors, AZD7762, PF477736, and LY2603618, upon BRD7 silencing and CHK1 accumulation (Fig. 5B, lanes 5 versus 1), in a dose-dependent manner (Figs. 5A and S5A). More specifically, the CCK8 cell viability assay showed that BRD7 silencing caused a significant decrease in the IC_50_ values of AZD7762, PF477736, and LY2603618, in H1650 cells, from 1.3 μΜ to 0.03 μΜ, from 17.6 μΜ to 2.4 μΜ, and from 8.8 μΜ to 0.5 μΜ, respectively (Fig. 5A). Mechanistically, CHK1 inhibitor treatment combined with BRD7 knockdown caused increased apoptosis, as reflected by the increased percentage of Annexin V^+^ cells (Fig. S5B), as well as increased cleavage of PARP and caspase-3, two hallmarks of apoptosis (Fig. 5B, lanes 6–8 versus 2–4; and S5C). Interestingly, upon BRD7 knockdown, CHK1 phosphorylation at Ser345 was moderately increased (Fig. 5B, lanes 5 versus 1), which was further dramatically amplified by the treatment of CHK1 inhibitors (Fig. 5B, lanes 6–8 versus 2–4; and S5C). However, the levels of total CHK1 were significantly decreased upon CHK1 inhibitor treatment (Fig. 5B, lanes 2–4 versus 1; and S5C), which may be caused by the increased ubiquitin-proteasome-dependent degradation induced by phosphorylation of CHK1 at Ser345 [44, 45]. This reduction was partially inhibited by BRD7 knockdown (Fig. 5B, lanes 6–8 versus 2–4). These results suggest that the increased level of total and phosphorylated CHK1 by BRD7 knockdown may contribute to increased sensitivity to CHK1 inhibitors. Indeed, simultaneous CHK1 knockdown completely reversed chemosensitization to CHK1 inhibitors caused by BRD7 deficiency (Fig. 5C). Interestingly, either knockdown BRD7 alone or simultaneous silencing of both BRD7 and CHK1 had no effects on the sensitivity to other genotoxic agents, including camptothecin (CPT), TOP I inhibitor, and etoposide (VP-16), TOP II inhibitor (Fig. S6). Taken together, these results indicate that BRD7 deficiency sensitizes tumor cells to CHK1 inhibitors, and CHK1 accumulation by BRD7 deficiency plays a causal role in promoting chemosensitization.

### BRD7 deficiency sensitizes tumor cells to CHK1 inhibitors in a USP1-dependent manner

**Fig. 6:**
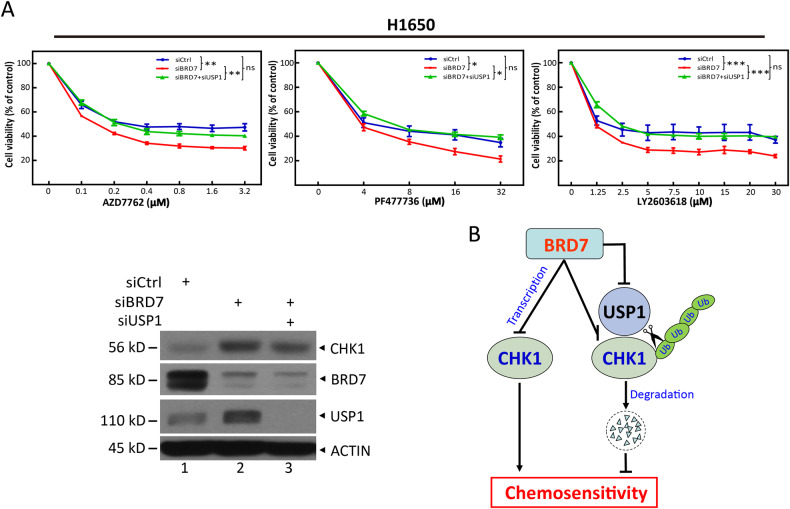
BRD7 deficiency sensitizes tumor cells to CHK1 inhibitors in a USP1-dependent manner. **A** H1650 cells transfected with the indicated siRNAs were plated in triplicate in 96-well plates, treated with various concentrations of AZD7762, PF477736, and LY2603618 for 72 h, and then subjected to CCK8 assay. The cells remaining in the plates were subjected to IB with the indicated antibodies (bottom). Data from three independent experiments are expressed as mean ± SEM; *p < 0.05, **p < 0.01, ***p < 0.001; ns: not significant. **B** A model for BRD7-USP1-mediated degradation of CHK1, which promotes tumor cell survival in response to chemotherapeutic drugs targeting CHK1.

As BRD7 regulates CHK1 stability and ubiquitination in a USP1-dependent manner, we next investigated whether simultaneous USP1 knockdown could repudiate the BRD7 deficiency-induced increased chemosensitization to CHK1 inhibitors. Indeed, simultaneous USP1 knockdown reversed the BRD7 deficiency-induced chemosensitization to CHK1 inhibitors (Fig. 6A), as well as increased protein level of CHK1 (Fig. 6A, lanes 3 versus 2). This further confirmed that BRD7 deficiency induced-CHK1 accumulation through USP1 plays a causal role in promoting chemosensitization.

## Discussion

CHK1 plays a crucial role in maintaining genome stability and the dysregulation of CHK1 may lead to abnormal cell cycle progression and tumorigenesis. Many cancer types are characterized by overexpression of CHK1, which increases the resistance of cancer cells to cancer therapy. These characteristics make CHK1 a potent molecular target for inhibition [46], and CHK1 inhibitors have been developed as anticancer agents. In our study, we found that BRD7 negatively regulated CHK1 levels in a variety of tumor cells and shortened CHK1 half-life by binding to and promoting CHK1 ubiquitination. Furthermore, we determined that regulation of CHK1 ubiquitination by BRD7 is dependent on USP1. Biologically, the BRD7-USP1-CHK1 axis regulates the sensitivity of tumor cells to chemotherapeutic drugs targeting CHK1, suggesting BRD7 as a new potential molecular target for effective anti-cancer therapy.

As the abnormal expression of CHK1 is associated with tumorigenesis and tumor cells with abundant expression of CHK1 are resistant to chemotherapy [47], it is important to determine the mechanisms and identify molecular targets that lead to excess CHK1 accumulation in tumor cells. Here, we found that BRD7 regulates CHK1 expression at both the mRNA and protein levels (Fig. 1A, B). Although p53 has been reported to be a transcriptional repressor of CHK1 [8], it does not participate in BRD7-mediated transcriptional regulation of the *CHK1* gene. The deficiency of p53 did not affect the *CHK1* gene expression induced by BRD7 silencing (Fig. 1C). Other transcriptional activators of CHK1, such as E2F1 and E4F1, negatively regulated CHK1 levels (Fig. S1C), which is contrary to the previously published reports [8, 37], suggesting a possible context-dependent regulatory function of E2F1 and E4F1. However, the specific transcription factors involved in the BRD7-mediated transcriptional regulation of CHK1 need to be investigated in future studies.

In addition, the overall activity of CHK1 can be regulated by post-translational modifications, including ubiquitination and phosphorylation, which are mainly responsible for the degradation and activation of CHK1. Our data show that BRD7 silencing increased the stability of CHK1 (Fig. 2A) and induced its phosphorylation at residues Ser^345^ (Fig. 5B, lanes 5 versus 1), leading to CHK1 activation, which suggests that BRD7 may be a novel factor that modulates CHK1 activity. Mechanistically, among the multiple E3s (CRL4^CDT2^, SCF^β-TrCP^, and SCF^FBXO6^) and deubiquitinases (USP1, USP7, and ATXN3), that have been reported to regulate CHK1 ubiquitination, only USP1 was correspondingly regulated in the case of BRD7 silencing. USP1 silencing reversed the regulatory effect of BRD7 on CHK1, suggesting that USP1 serves as a mediator for BRD7 regulation of CHK1. Furthermore, the expression of the two E3 ligases, CDT2 and β-TrCP, was increased by BRD7 silencing. However, CDT2 and β-TrCP did not decrease the expression of CHK1 in BRD7 knockdown cells, suggesting that other complementary mechanisms may be involved in simultaneously maintaining CHK1 levels, which reversed the inhibitory effect of CDT2 and β-TrCP on CHK1.

Several chemotherapeutic drugs targeting CHK1 overexpression have been developed and used as single agents or paired with anticancer drugs in clinical studies, such as AZD7762, LY2603618, MK-8776, and PF477736 [48]. These genotoxic drugs can effectively inhibit cell proliferation and induce apoptosis in cells with high expression and activity of CHK1. However, use of many CHK1 inhibitors has been terminated in clinical trials owing to their cellular or tissue toxicity or other side effects [48], which may be partially overcome by increasing the sensitivity of tumor cells to CHK1 inhibitors. Our data shows that the increased expression and activity of CHK1 induced by BRD7 silencing could be a promising strategy to enhance the chemosensitivity of tumor cells to CHK1 inhibitors. Under BRD7 deficiency and CHK1 accumulation, the CHK1 inhibitors induced higher tumor cell apoptosis (Figs. 5B and S5B), with improved efficiency in killing tumor cells and reduced side effects.

In summary, our study uncovered BRD7 as a novel negative regulator of CHK1 expression. First, BRD7 inhibited the transcription of CHK1. Second, BRD7 negatively regulated the transcription of USP1, a deubiquitinase of CHK1. Furthermore, BRD7 inhibited USP1 binding to CHK1, leading to CHK1 ubiquitination and proteasomal degradation. As a result, BRD7 suppressed chemosensitivity by eliminating CHK1 (Fig. 6B). Therefore, BRD7 may be a potential genetic or drug target for improving the efficacy of chemotherapeutic drugs targeting CHK1 in future cancer therapies.

## Materials and methods

### Cell lines and chemicals

A549, HEK293, H358, H1650, H1792, HCT116, MCF7, SK-BR3, and U2OS cells were obtained from American Type Culture Collection (ATCC). Cells for experiments were passaged for fewer than 25–30 times. A549, HEK293, H1792, MCF7, SK-BR3, and U2OS cells were maintained in Dulbecco’s modified Eagle’s medium (DMEM) supplemented with 10% (v/v) fetal bovine serum (FBS). H358 and H1650 cells were maintained in Roswell Park Memorial Institute (RPMI) 1640 medium supplemented with 10% FBS. HCT116 cells were maintained in McCoy’s 5A medium containing 10% FBS. HCT116 *p53*^*+/+*^ and *p53*^*−/−*^ cells were kindly provided by Professor Bert Vogelstein. U2OS *p53*^*+/+*^ and *p53*^*−/−*^ cells were generated using CRISPR/Cas9 technology as previously described [49]. The following chemicals were obtained from commercial sources: CHX (C7698; Sigma), MG132 (10012628; Cayman), AZD7762 (S1532; Selleck), PF477736 (S2904; Selleck), and LY2603618 (S2626; Selleck).

### Immunoblotting (IB) and immunoprecipitation (IP)

For IB, whole cells were lysed in lysis buffer [50 mM Tris-HCl (pH 7.5), 0.15 M NaCl, 1% NP-40, 0.1% SDS, 0.5% Sodium Deoxycholate, 50 mM NaF, 1 mM Na_3_VO_4_, 1 mM EDTA, 1 mM DTT] with protease and phosphatase inhibitors, followed by supernatant harvesting. Protein concentration was measured using the BCA protein assay kit (23225; Thermo). The cell lysates were then separated by sodium dodecyl sulfate-polyacrylamide gel electrophoresis (SDS-PAGE) and immunoblotted with the indicated antibodies [50]. For IP, to determine BRD7 binding with CHK1, cells transfected with FLAG-CHK1 (exogenous) or whole cells (endogenous) were lysed and then incubated with bead-conjugated anti-FLAG or CHK1 antibody in a rotating incubator for 3–5 h at 4 °C. Then, the cell lysates were incubated with Protein G Sepharose beads for an additional 4 h. The immunoprecipitates were washed with lysis buffer and subjected to IB.

The following antibodies were used: CHK1 (sc-8408; Santa Cruz; 1:1000), BRD7 (15125; Cell Signaling Technology; 1:1000), CHK2 (6334; Cell Signaling Technology; 1:1000), ACTIN (A5441; Sigma; 1:10000), p53 (OP43; Calbiochem; 1:1000), E4F1 (ab70615; Abcam; 1:5000), E2F1 (3742; Cell Signaling Technology; 1:1000), FLAG (F1804; Sigma; 1:2000), HA (3724; Cell Signaling Technology; 1:5000), USP1 (8033; Cell Signaling Technology; 1:10000), USP7 (4833; Cell Signaling Technology; 1:20000), ATXN3 (3467813; Millipore; 1:10000), CDT2 (A300-948A; Bethyl; 1:1000), β-TrCP (4394; Cell Signaling Technology; 1:1000), FBXO6 (sc-134339; Santa Cruz; 1:500), p-CHK1 (Ser345) (2348, Cell Signaling Technology, 1:1000), PARP (9542; Cell Signaling Technology, 1:1000), and caspase 3 (9665; Cell Signaling Technology, 1:1000).

### Quantitative RT-PCR (RT-qPCR)

Total RNA was isolated from cells using TRIzol reagent (15596018; Invitrogen). cDNA was synthesized from RNA via reverse transcription using the PrimeScript RT reagent kit (RR037A; Takara). RT-qPCR performed using SYBR® Premix Ex Taq™ (RR420A; Takara) on an Applied Biosystems Viia7 real-time PCR system. The primers used for qRT-PCR were as follows: CHK1 forward: 5′-CTG CAA TGC TCG CTG GAG AAT-3′, reverse: 5′-GGG CTG GTA TCC CAT AAG GAA AGA-3′; CHK2 forward: 5′-GGC TCA CGC GGT CGT-3′, reverse: 5′-GAG CCT TGG GAC TGG GTA AC-3′; BRD7 forward: 5′-TGG AGA TGT CAT TGC CTG AAG A-3′, reverse: 5′-CCC TGG TGG CTC TAC TTC TG-3′; GAPDH forward: 5′-AGG GCA TCC TGG GCT ACA C-3′, reverse: 5′-GCC AAA TTC GTT GTC ATA CCA G-3′.

### siRNA, shRNA silencing and plasmid overexpression

Cells were transfected with the following siRNA oligos using Lipofectamine 2000. siCtrl: 5′-ATT GTA TGC GAT CGC AGA C-3′; siBRD7#1: 5′-GCC AAG ATT ATC CGT ATG TCA-3′; siBRD7#2: 5′-GTA CTA ATG CCA TGA TTT A-3′ (siBRD7 labled in this manuscript were siBRD7#2); siE2F1#1: 5′-GAG GAG TTC ATC AGC CTT T-3′; siE2F1#2: 5′-AAA GTT CTC CGA AGA GTC CAC GGC T-3′; siE4F1#1: 5′-GAA GCC GTT CAA GTG CTA CAA-3′; siE4F1#2: 5′-GAC AGG GCA GAG GAC TCT GAA-3′; siUSP1: 5′-GCT CGT ATT TGT ATT CTC CAT-3′; and siCHK1: 5’-TCG TGA GCG TTT GTT GAA C-3’. The BRD7-targeting shRNA, 5′-GTA CTA ATG CCA TGA TTT A-3′, was cloned into the pLKO.1-puro vector. Lentiviral shRNA virus packaging and subsequent infection of cells were performed as previously described [51]. 3 × FLAG-tagged human BRD7 and CHK1 cDNA were subcloned into pIRES2-EGFP. HA-tagged human CHK1 and 6 × HIS-tagged Ub were subcloned into pcDNA3.1.

### In vivo ubiquitination assay

HEK293 cells infected with indicated shRNA were transfected with the indicated plasmids or/and siRNA oligos for 48 h and then treated with 20 µM MG132 for 4–6 h. Cells were then lysed in a 6 M guanidine denaturing solution and incubated with Ni-NTA agarose (1018244; Qiagen), as described previously [51].

### Flow cytometry

For cell cycle analysis, cells transfected with the indicated siRNAs were collected by trypsinizing gently and fixed in 70% ethanol overnight at −20°C. Cells were stained with Propidium Iodide (550825; BD Biosciences) for 15 min and analyzed by flow cytometry.

For cell apoptosis analysis, cells transfected with the indicated siRNAs were treated with AZD7762, PF477736, and LY2603618 respectively for 24 h, followed by staining with Annexin V-FITC/propidium iodide (PI) using an apoptosis detection kit (C1062; Beyotime) according to the manufacturer’s instructions. Subsequently, the cells were analyzed by flow cytometry.

### CCK8 assay

Cells were transfected with indicated siRNA oligos for 36 h and then seeded in triplicate in 96-well plates. After 24 h, cells were treated with AZD7762, PF477736, and LY2603618 at various concentrations for 72 h and then assayed for viability using the Cell Counting kit-8 (MedChemExpress) according to the manufacturer’s instructions. For cell proliferation, cells were seeded in 96-well plates in triplicate at 3000 cells per well and then assasyed each day using Cell Counting kit-8. The data were obtained from three independent experiments, each run in triplicate.

### Clonogenic survival assay

A total of 300 cells (for A549) or 250 cells (for H1650) were seeded in 60-mm dishes pretreated with gelatin. After 10–14 days, the cells were fixed and stained with Coomassie brilliant blue solution. The colonies were counted over 50 cells per colony and photographed by Bio-Rad imaging system.

### EdU incorporation assay

DNA replication was determinded by incorporation of 5-ethynyl-2′-deoxyuridine (EdU) using BeyoClick™ EdU Cell Proliferation Kit with Alexa Fluor 488 (C0071S) according to the manufacturer’s instructions. Briefly, cells were seeded in 24-well plates the day before, then treated with 10 μM EdU working solution for 2 h. After EdU labeling, cells were incubated with fixative medium, permeabilization solution, and click reaction solution in order. The cells were photographed under a fluorescence microscope.

### Statistical analysis

Data from three independent biological replicates were presented as the mean ± SEM. Statistical analyses between two groups were determined by two-tailed Student’s *t*-test and statistical analyses from more than two groups were determined by ANOVA using GraphPad Prism 8. Differences were considered statistically significant at *p* < 0.05.

### Supplementary information

Supplemental information

Supplemental Figure 1

Supplemental Figure 2

Supplemental Figure 3

Supplemental Figure 4

Supplemental Figure 5

Supplemental Figure 6

Original IBs

Author Contribution Statement

## Data Availability

The authors declare that all data supporting the findings of this study are available with the article or from the corresponding author upon reasonable request.
