# Supplementary material for: BRD7 suppresses tumor chemosensitivity to CHK1 inhibitors by inhibiting USP1-mediated deubiquitination of CHK1
Source: Cell Death Discov. 2023 Aug 25;9:313. doi: 10.1038/s41420-023-01611-x (PMC10457387; doi:10.1038/s41420-023-01611-x)
Supplement: Supplementary file 1 — Supplemental information [file 41420_2023_1611_MOESM1_ESM.docx]

**Supplemental information**

**Supplemental figure legends**

**Figure S1. BRD7 negatively regulates CHK1 expression in tumor cells.**

**A-B,** Silencing of BRD7 increases CHK1 levels but not CHK2. Various tumor cells transfected with siRNA targeting BRD7 (siBRD7) or scrambled control siRNA (siCtrl) were harvested at 48 h, followed by immunoblotting (IB) with the indicated antibodies. **C,** The transcription factors E4F1 and E2F1 negatively regulate CHK1 expression. Cells transfected with two different siRNAs targeting E4F1 (siE4F1) or E2F1 (siE2F1) or scrambled control siRNA (siCtrl) were harvested at 48 h, followed by IB with the indicated antibodies.

**Figure S2. Proteasome inhibitor inhibits the decreased CHK1 by BRD7 overexpression.**

HEK293 cells were transfected with the indicated plasmids for 48 h and then treated with 20 µM MG132 for 6 h, followed by immunoblotting with the indicated antibodies. LEX: longer exposure.

**Figure S3. BRD7 negatively regulates the level of USP1 and the interaction between USP1 and CHK1.**

**A,** Silencing of BRD7 increases USP1 protein levels. U2OS cells transfected with siRNA targeting BRD7 or scrambled control siRNA were harvested for immunoblotting (IB) with the indicated antibodies. **B,** Silencing of USP1 shortens CHK1 half-life. H1650 cells transfected with the indicated siRNAs were treated with 100 µg/mL CHX for the indicated time periods and then subjected to IB with the indicated antibodies. **C,** Silencing of BRD7 enhances the interaction between USP1 and CHK1. A549 cells transfected with siRNA targeting BRD7 or scrambled control siRNA were lysed for IP with CHK1 antibody or normal IgG, followed by IB for the indicated antibodies. Whole-cell lysates (WCE) were subjected to IB with the indicated antibodies.

**Figure S4. BRD7 deficiency promotes DNA replication, cell proliferation, and survival in a CHK1-dependent manner.**

**A,** Silencing of BRD7 increases CHK1 levels and simultaneous silencing of BRD7 and CHK1 downregulates CHK1 levels. A549 cells transfected with indicated siRNA were harvested at 48 h, followed by immunoblotting (IB) with the indicated antibodies. **B,** Silencing of BRD7 increases DNA replication, while simultaneous silencing of BRD7 and CHK1 suppresses DNA replication. A549 cells transfected with indicated siRNA were subjected to EdU incorporation assay using BeyoClick™ EdU Cell Proliferation Kit. The percentage of EdU-positive cells in the total number of cells was determined from at least five random fields. **C-E,** Silencing BRD7 increases the percentage of S phase of cell cycle (**C**), cell proliferation (**D**) and cell survival (**E**) in a CHK1-dependent manner. A549 cells were transfected with the indicated siRNA oligos for 48 h and then split for flow cytometry (C), CCK8 assay (D), and clonogenic survival assay (E). Data from three independent experiments are expressed as mean ± SEM, *p < 0.05, **p < 0.01, ***p < 0.001; ns: not significant.

**Figure S5. BRD7 deficiency sensitizes tumor cells to CHK1 inhibitors.**

**A,** Silencing of BRD7 sensitizes cells to CHK1 inhibitors. A549 cells transfected with siRNA targeting BRD7 or scrambled control siRNA were plated in triplicate in 96-well plates and then treated with various concentrations of AZD7762, PF477736, and LY2603618 for 72 h for CCK8 assay. Data from three independent experiments were expressed as mean ± SEM, *p < 0.05, **p < 0.01, ***p < 0.001. **B-C,** Silencing of BRD7 promotes CHK1 inhibitor-induced cell apoptosis. A549 cells transfected with siRNA targeting BRD7 or scrambled control siRNA were treated with AZD7762, PF477736, and LY2603618 for 24 h (**B**) or the indicated times (**C**) and then subjected to fluorescence-activated cell sorting (FACS) analysis to determine the apoptotic population (**B**, left, representative FACS profiles; right, the percentage of Annexin V^+^ and Annexin V^+^/PI^+^ cells, mean ± standard error of the mean [SEM], n=3, **p < 0.01) or immunoblotting (IB) with the indicated antibodies (**C**). LEX: longer exposure.

**Figure S6. BRD7 deficiency has on effects on the sensitivity to genotoxic agents camptothecin (CPT) and etoposide (VP16).**

H1650 and A549 cells transfected with indicated siRNAs were plated in triplicate in 96-well plates and treated with various concentrations of CPT, and VP16 for 72 h for the CCK8 assay. Data from three independent experiments are expressed as mean ± SEM, ns: not significant.
