# Supplementary figures and images for: BRD7 suppresses tumor chemosensitivity to CHK1 inhibitors by inhibiting USP1-mediated deubiquitination of CHK1
Source: Cell Death Discov. 2023 Aug 25;9:313. doi: 10.1038/s41420-023-01611-x (PMC10457387; doi:10.1038/s41420-023-01611-x)

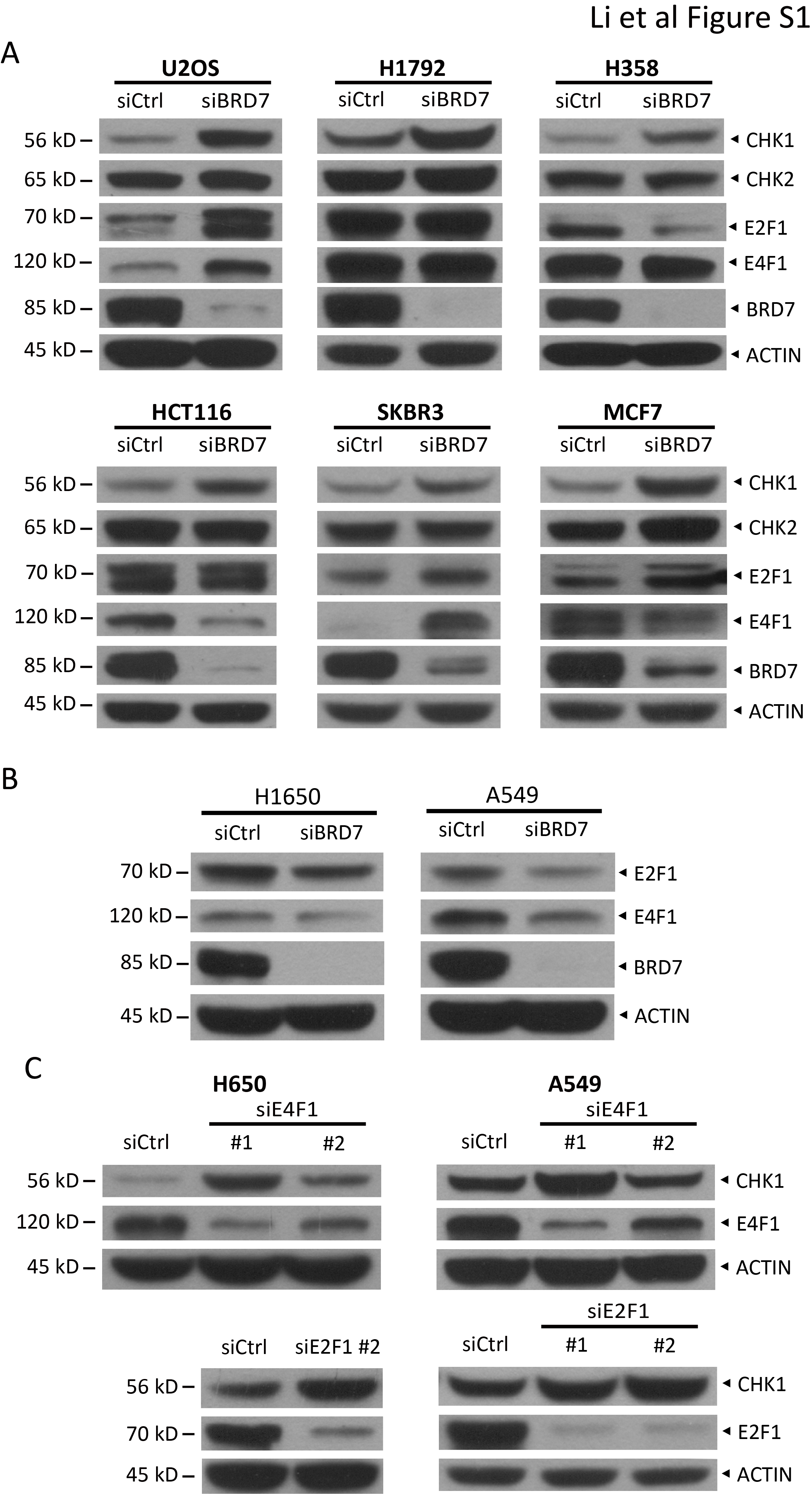

Supplement: Supplementary file 2 — Supplemental Figure 1 [file 41420_2023_1611_MOESM2_ESM.png]

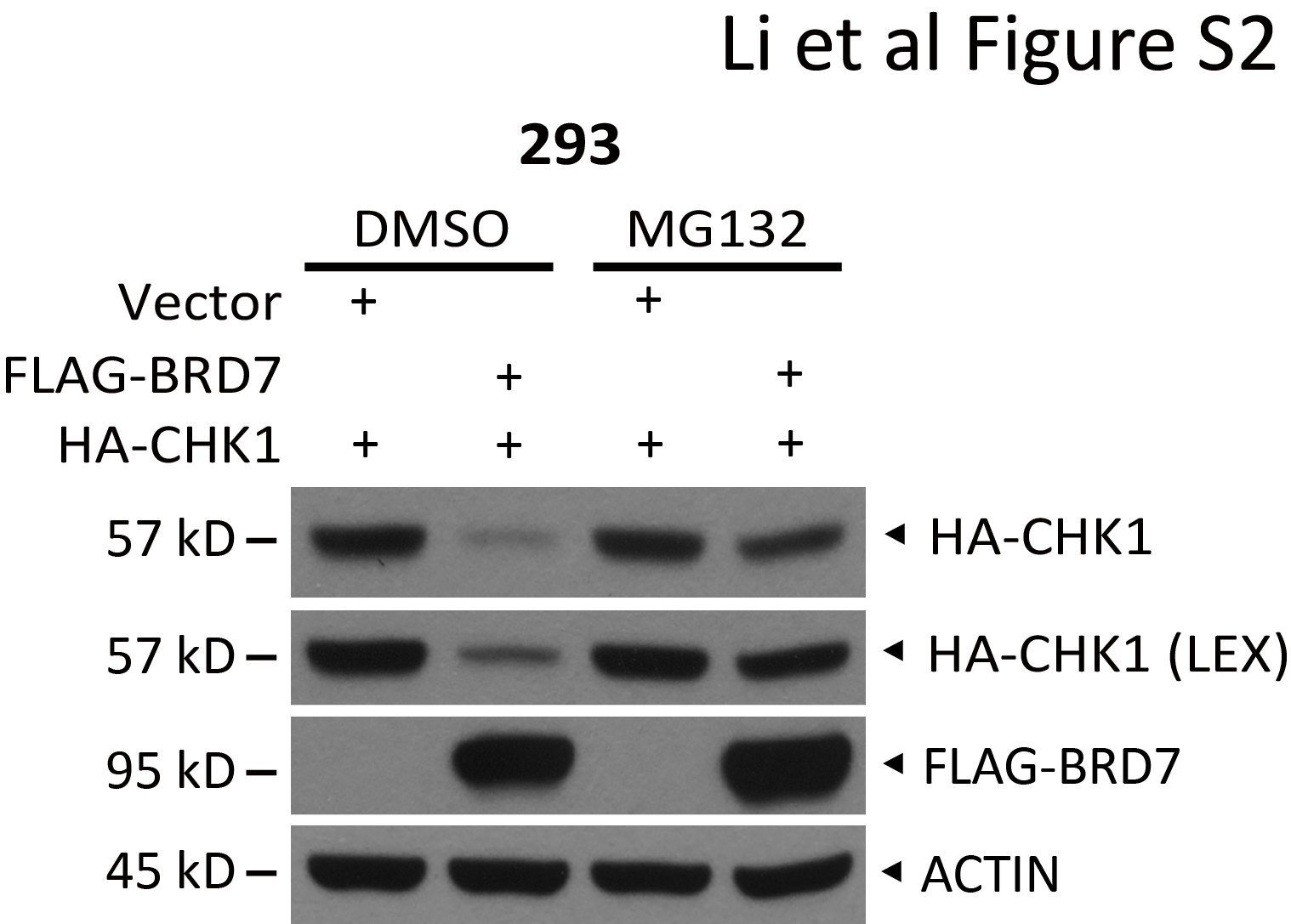

Supplement: Supplementary file 3 — Supplemental Figure 2 [file 41420_2023_1611_MOESM3_ESM.png]

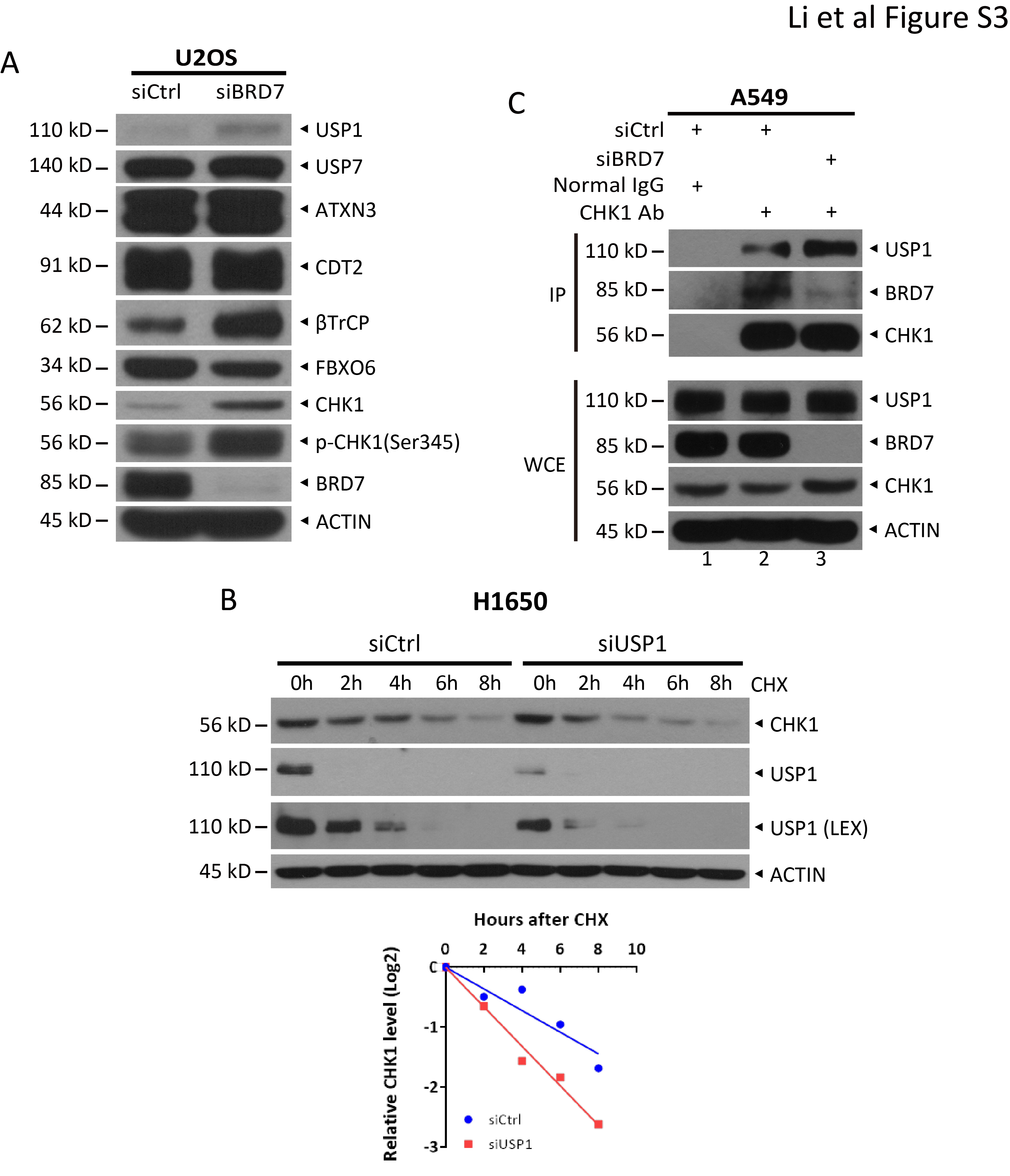

Supplement: Supplementary file 4 — Supplemental Figure 3 [file 41420_2023_1611_MOESM4_ESM.png]

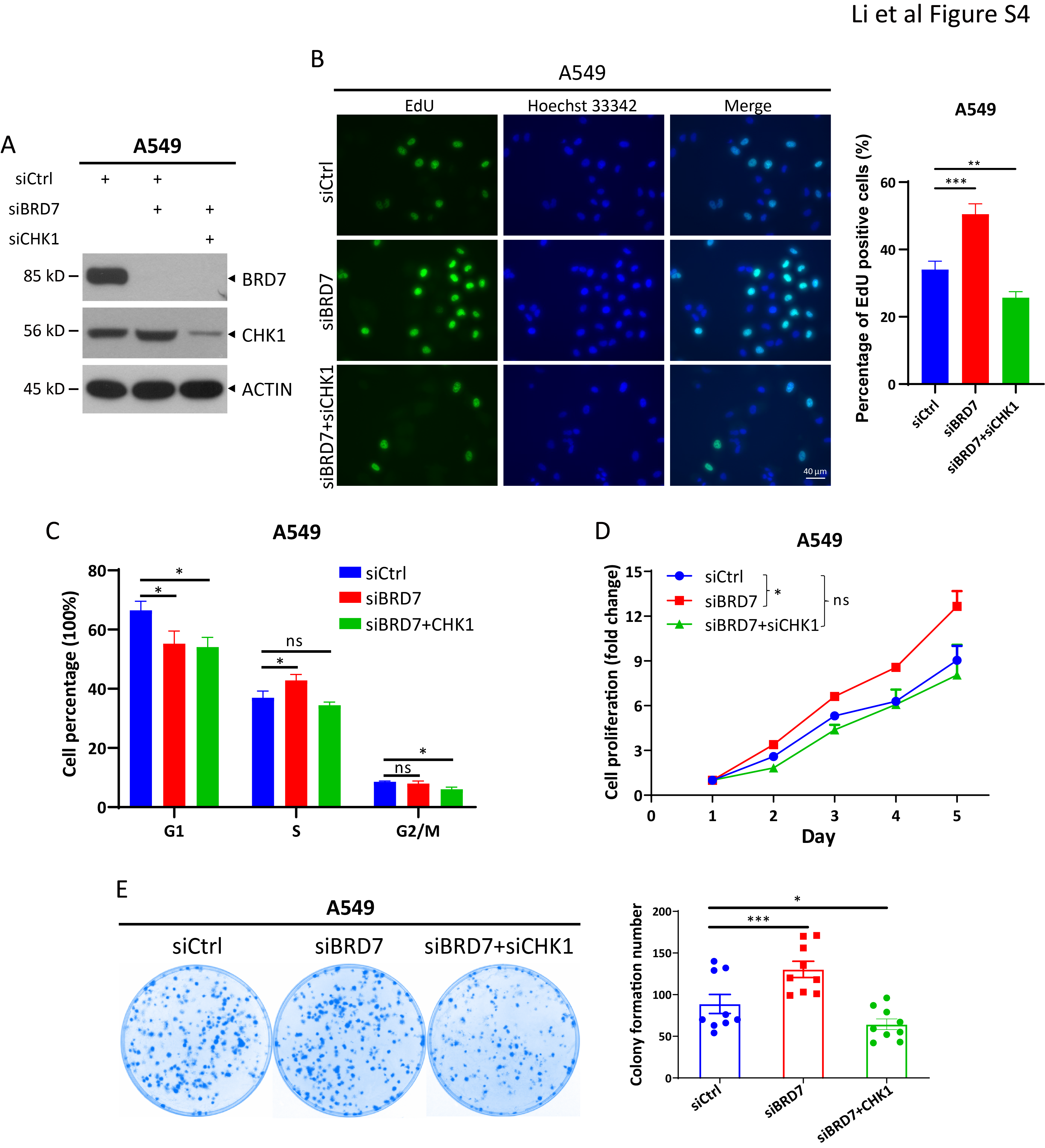

Supplement: Supplementary file 5 — Supplemental Figure 4 [file 41420_2023_1611_MOESM5_ESM.png]

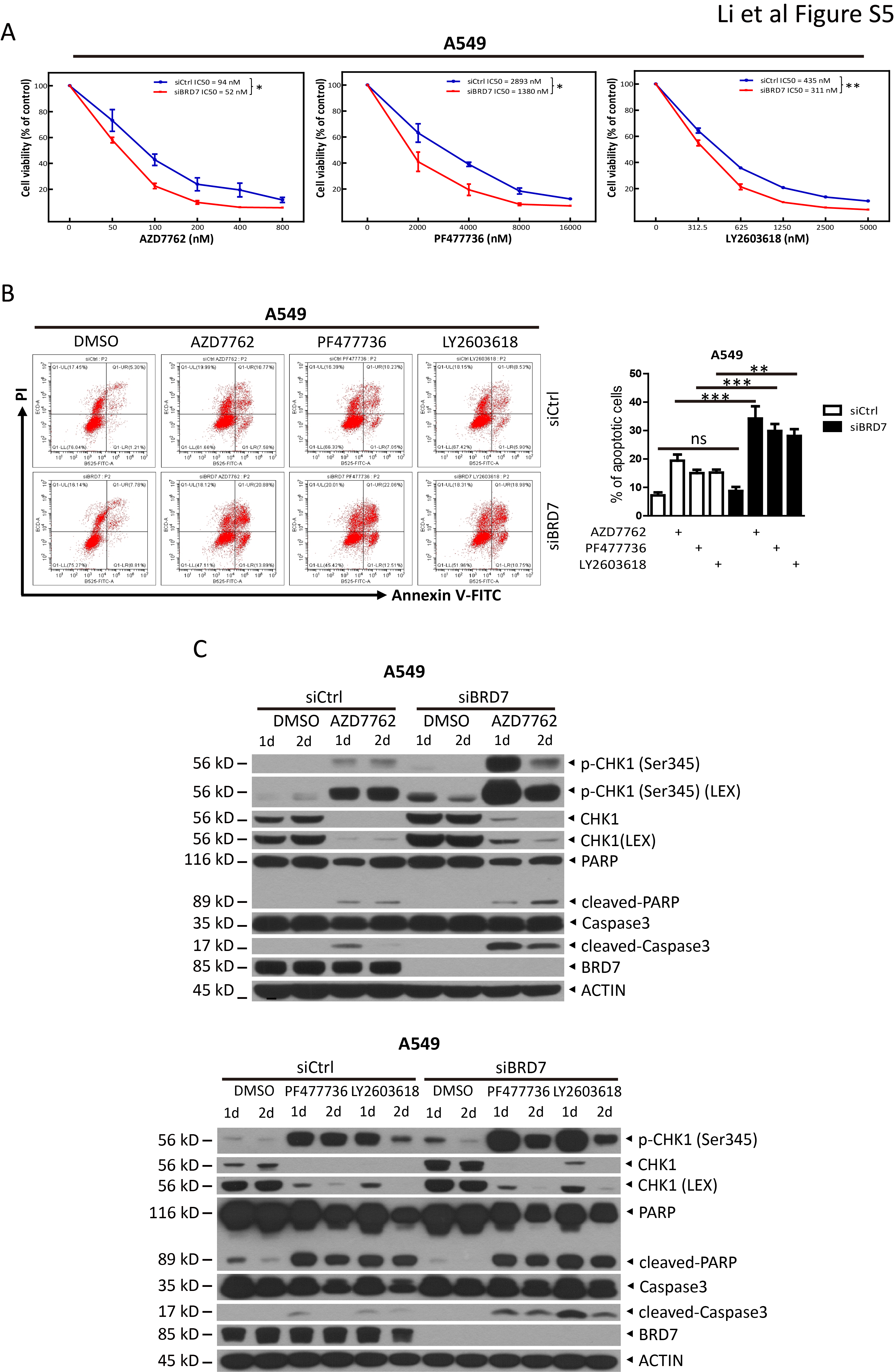

Supplement: Supplementary file 6 — Supplemental Figure 5 [file 41420_2023_1611_MOESM6_ESM.png]

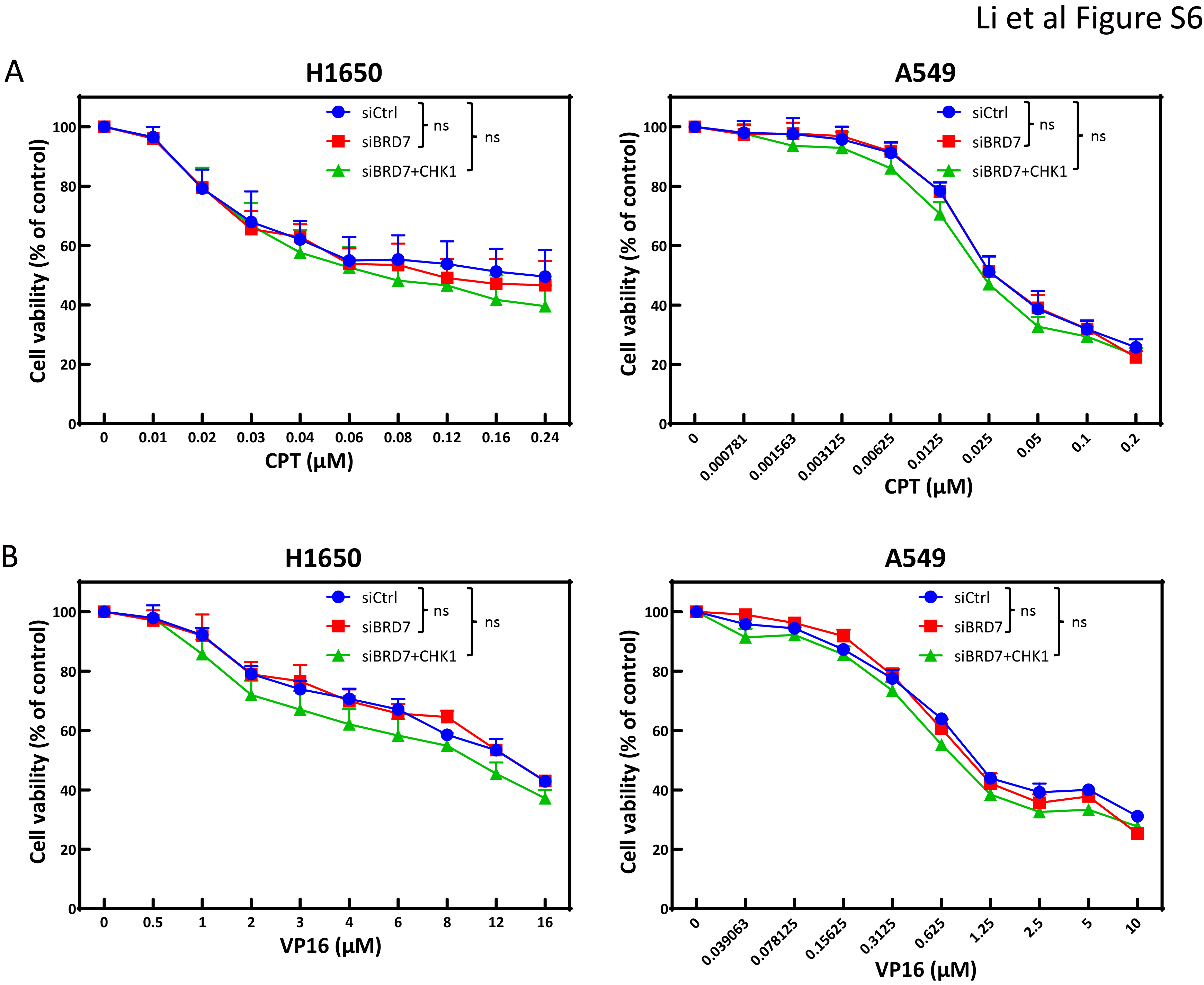

Supplement: Supplementary file 7 — Supplemental Figure 6 [file 41420_2023_1611_MOESM7_ESM.png]
