# Supplementary material for: BRD7 suppresses tumor chemosensitivity to CHK1 inhibitors by inhibiting USP1-mediated deubiquitination of CHK1
Source: Cell Death Discov. 2023 Aug 25;9:313. doi: 10.1038/s41420-023-01611-x (PMC10457387; doi:10.1038/s41420-023-01611-x)
Supplement: Supplementary file 8 — Original IBs [file 41420_2023_1611_MOESM8_ESM.pdf]

Figure 1A

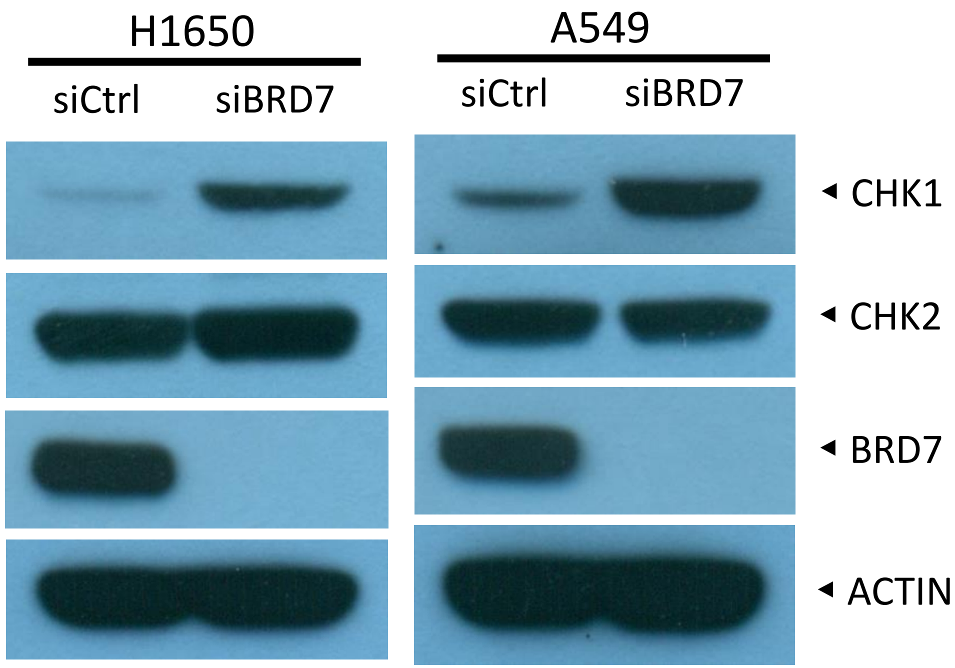

Figure 1C

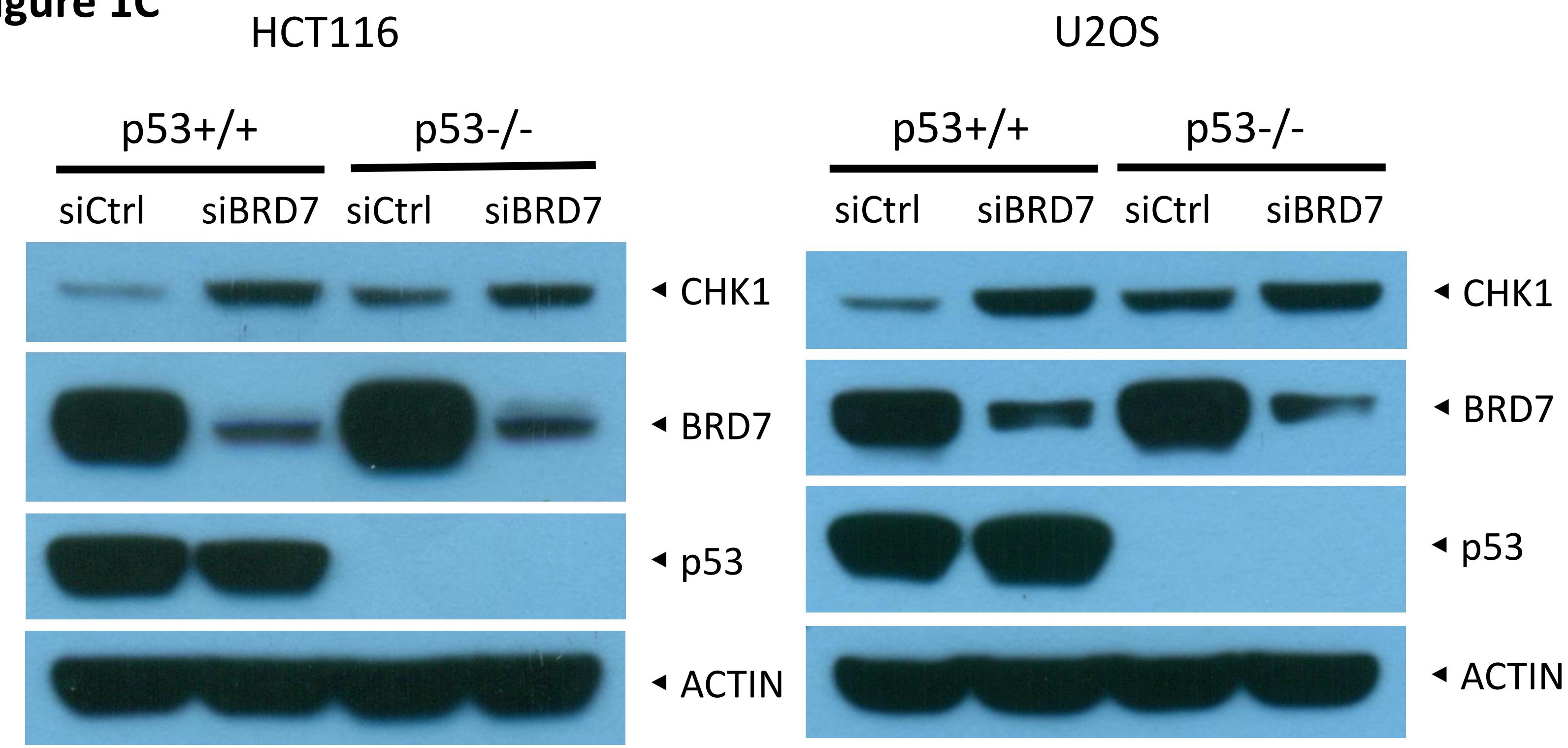

Figure 2A

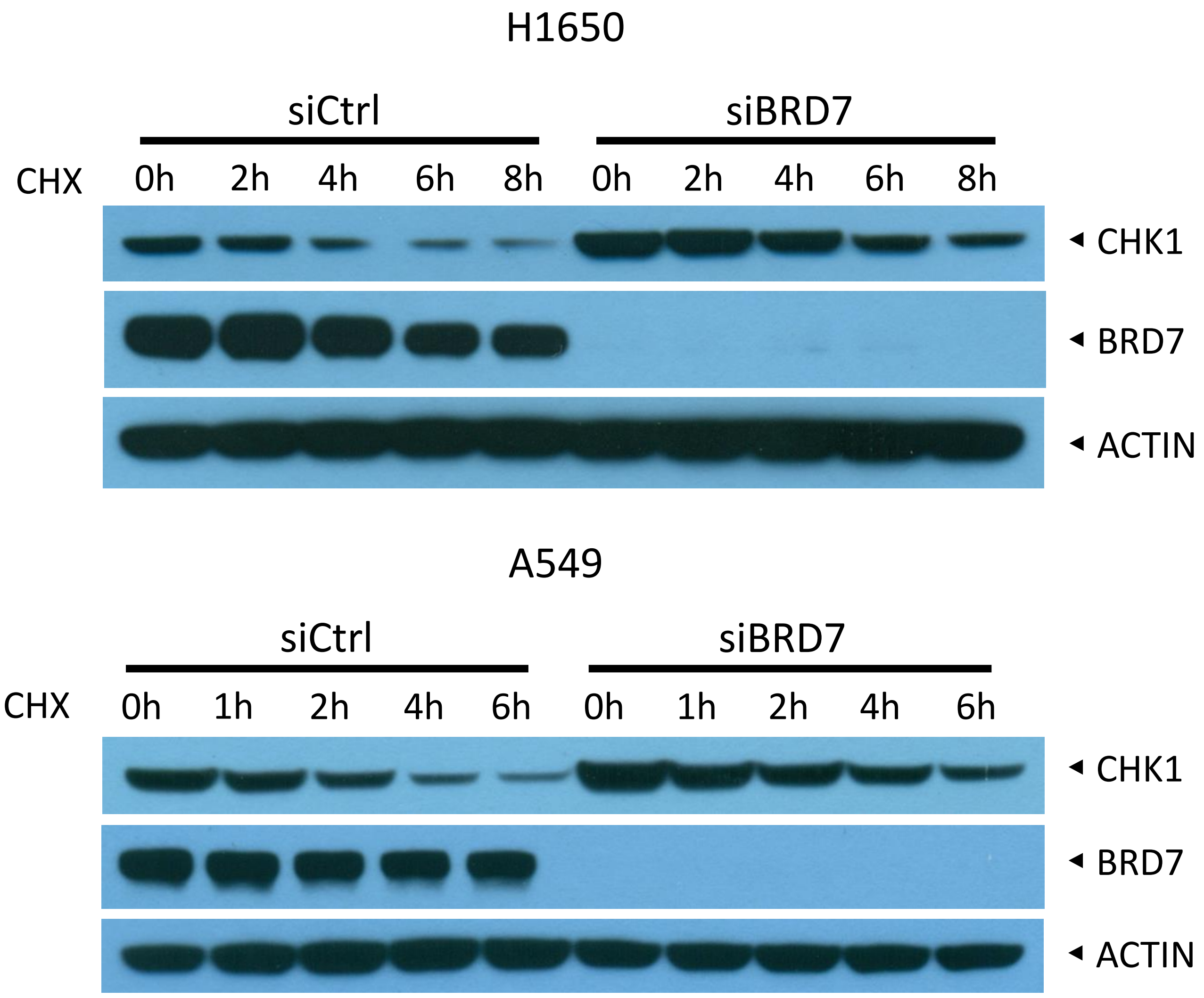

Figure 2B

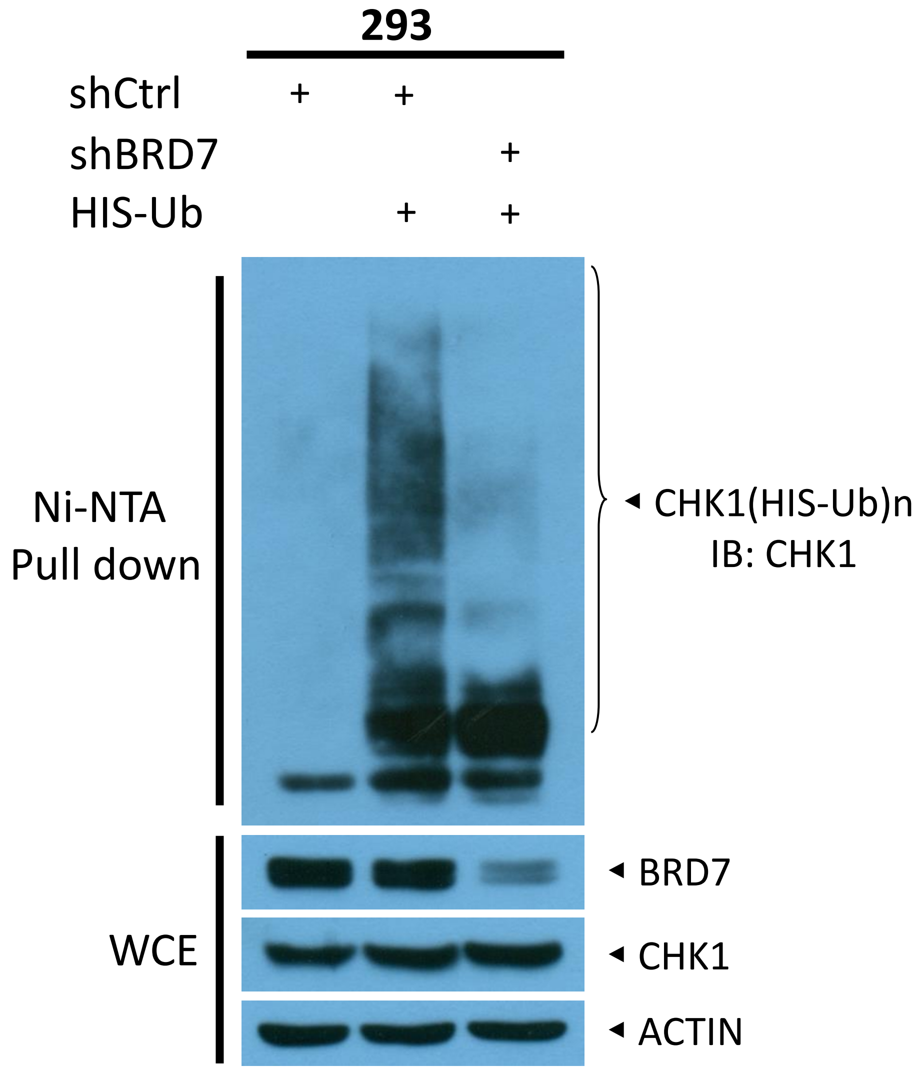

Figure 2C

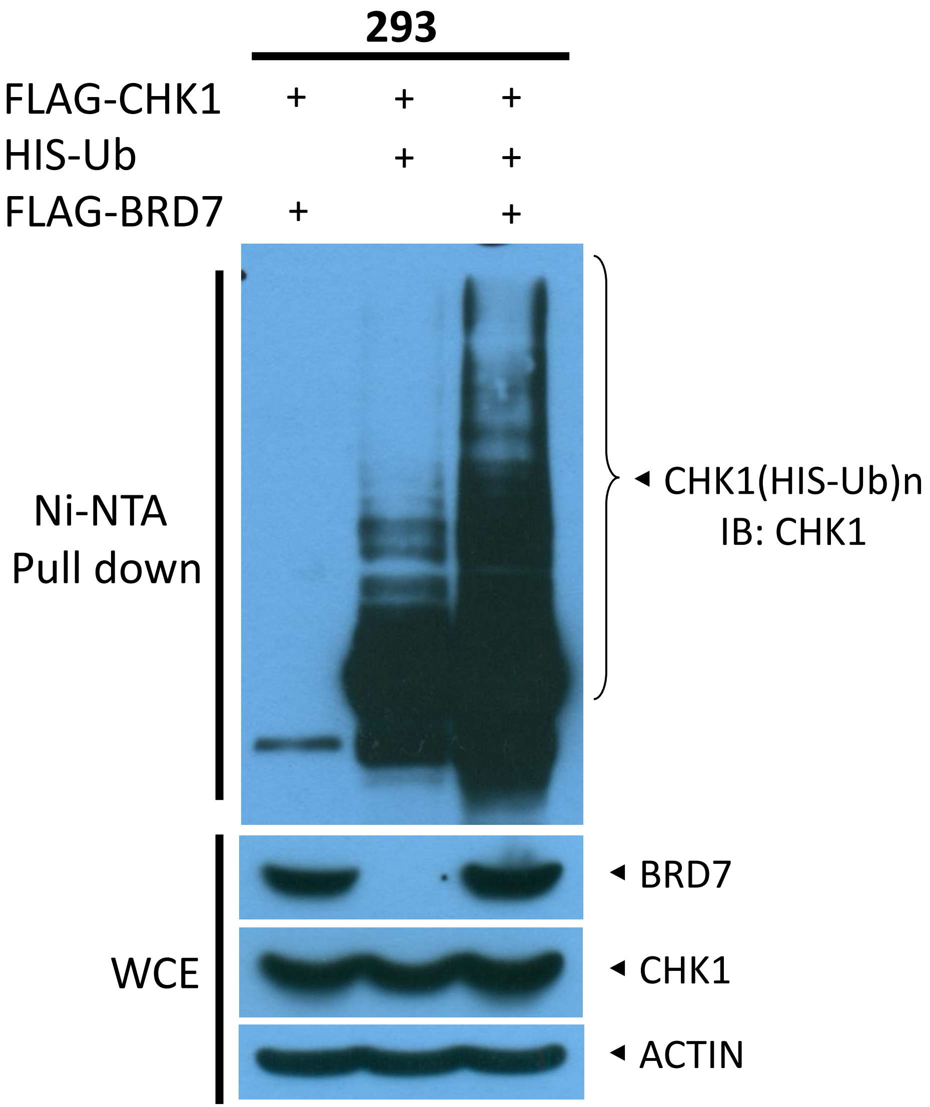

Figure 2D

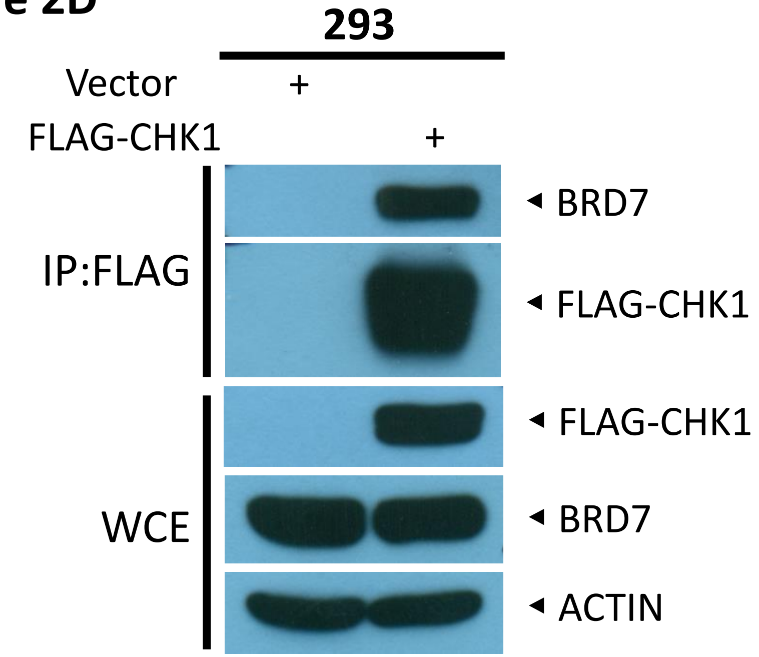

Figure 2E

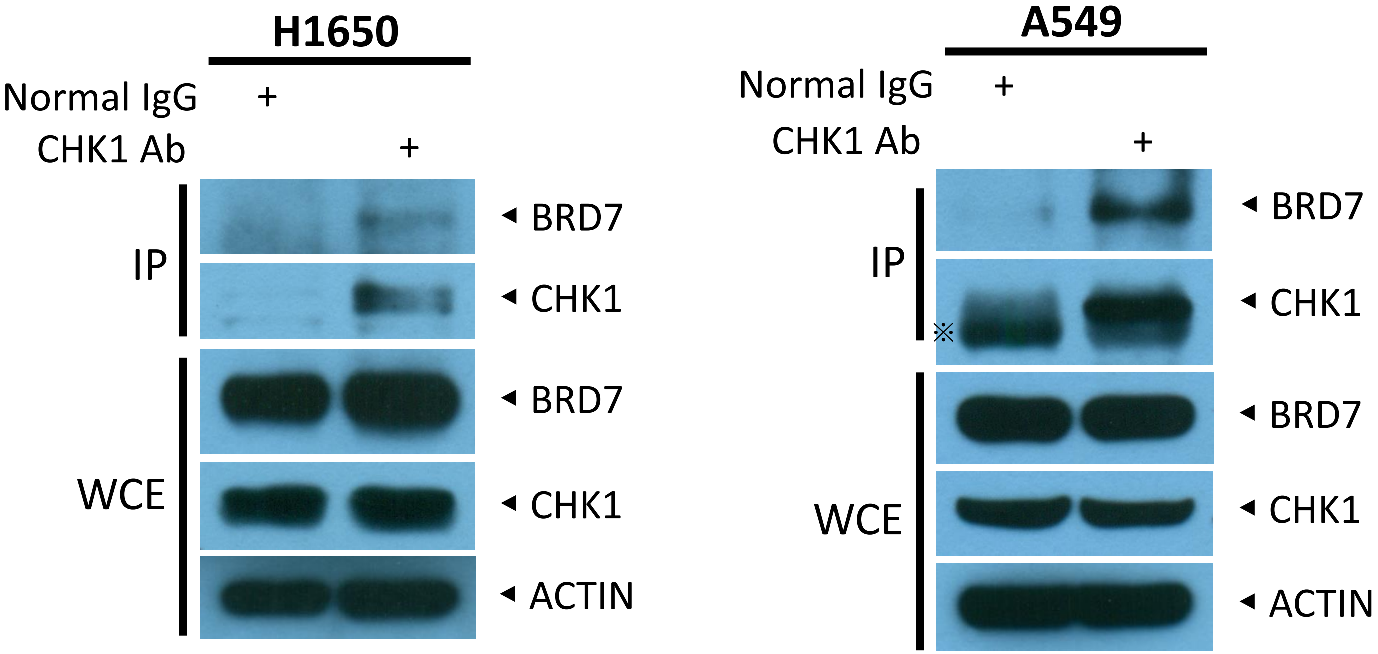

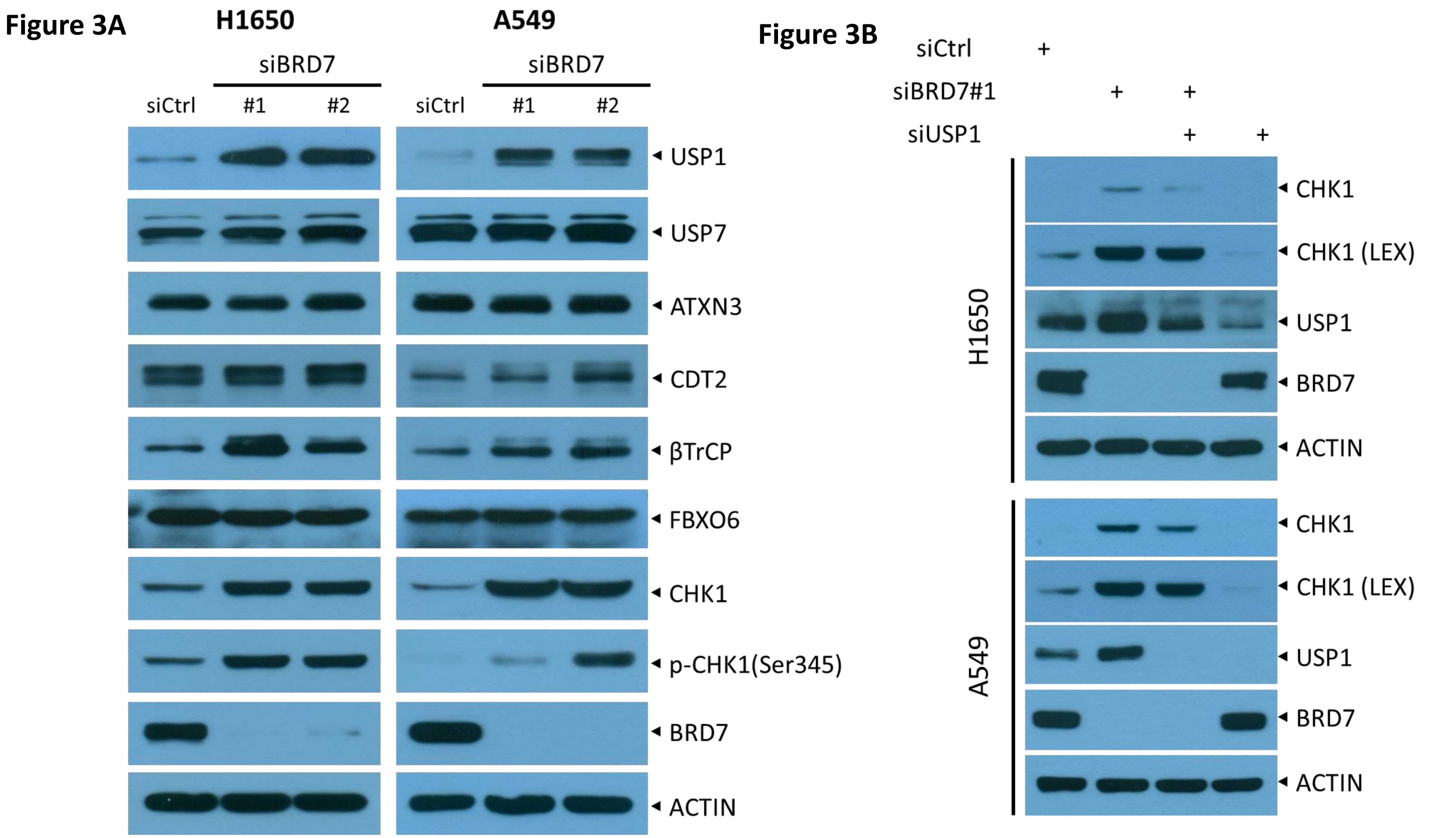

Figure 3A
H1650
A549
siBRD7
siCtrl
#1
#2
USP1
USP7
ATXN3
CDT2
βTrCP
FBXO6
CHK1
p-CHK1(Ser345)
BRD7
ACTIN
Figure 3B
siCtrl
siBRD7#1
siUSP1
+
H1650
A549
CHK1
CHK1 (LEX)
USP1
BRD7
ACTIN

Figure 3C

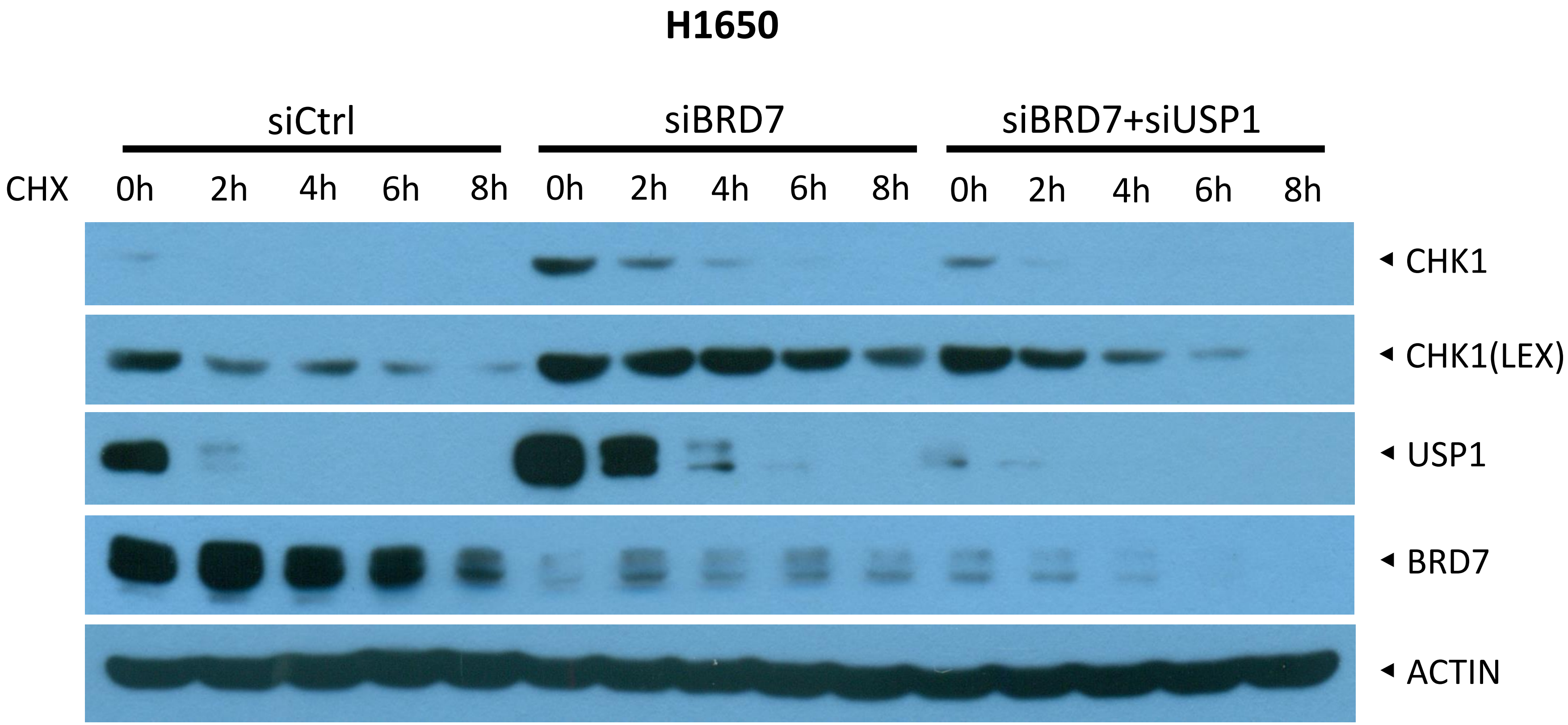
H1650
siCtrl
siBRD7
siBRD7+siUSP1
CHX
0h 2h 4h 6h 8h 0h 2h 4h 6h 8h 0h 2h 4h 6h 8h
CHK1
CHK1(LEX)
USP1
BRD7
ACTIN

Figure 3D

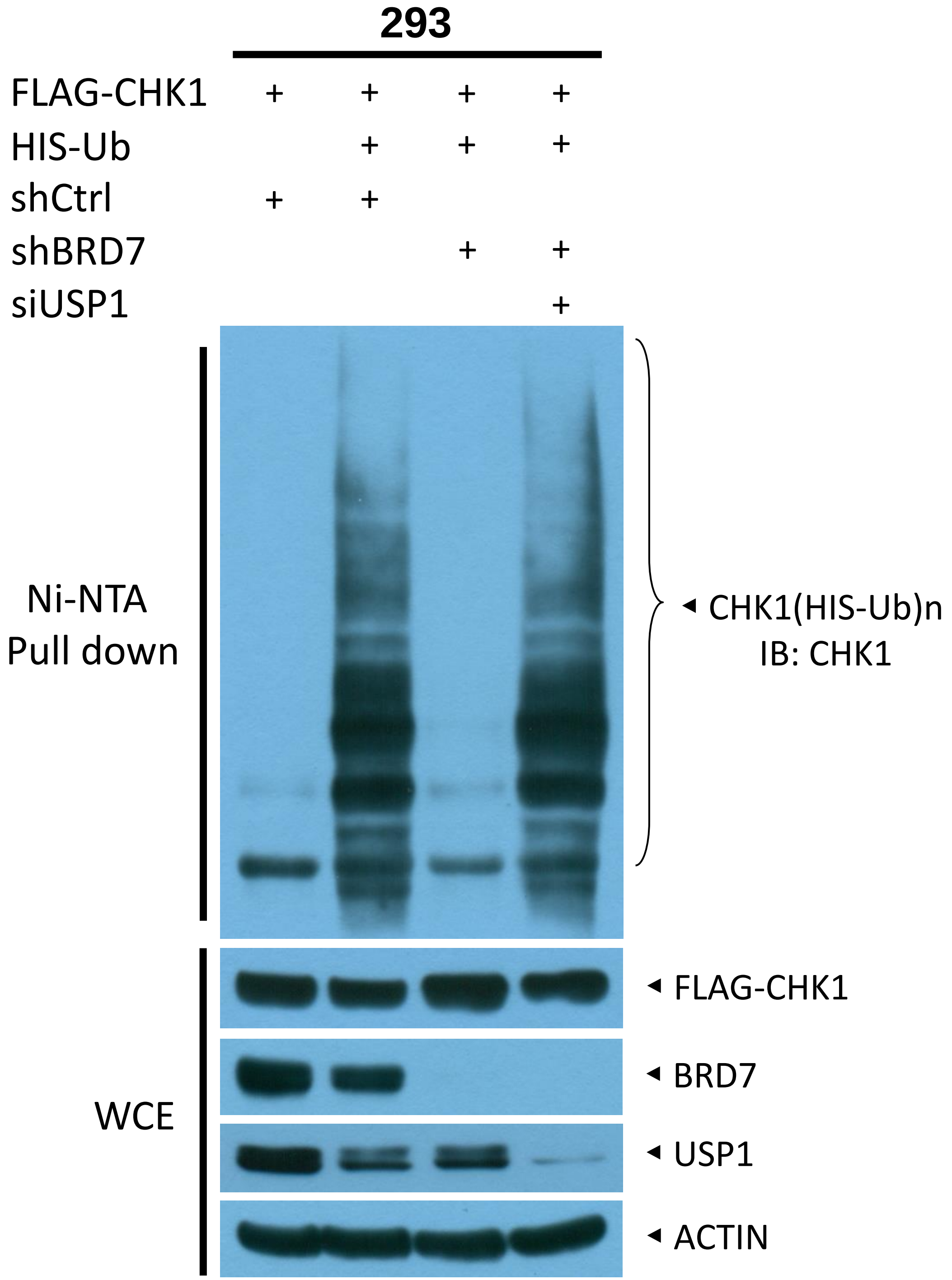

Figure 3E

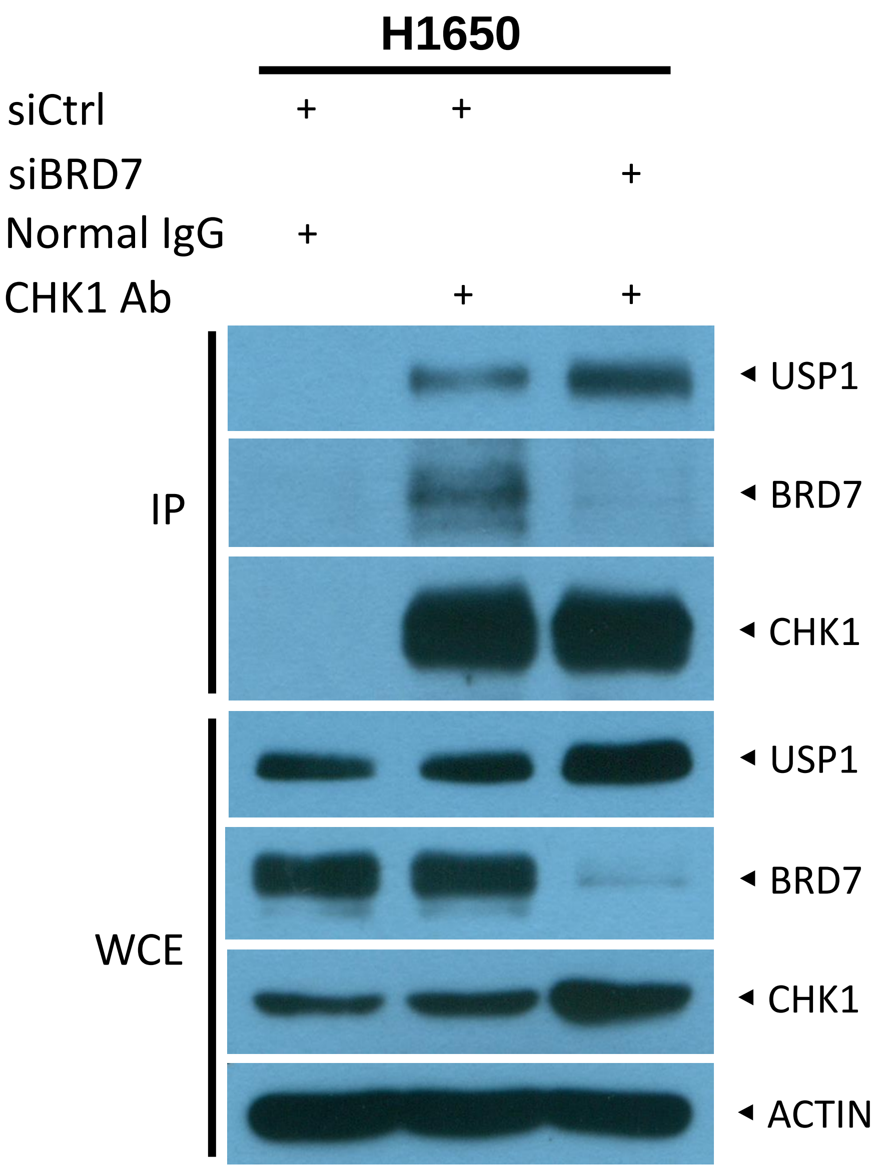

Figure 4A

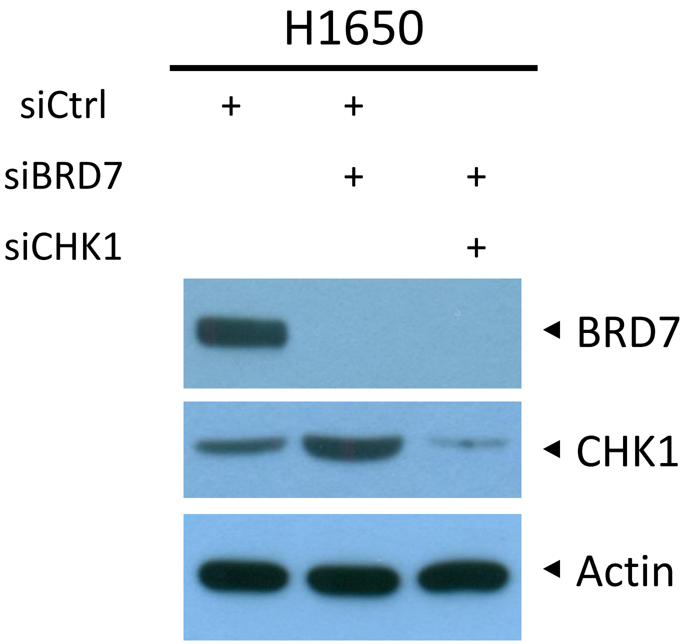

Figure 5B

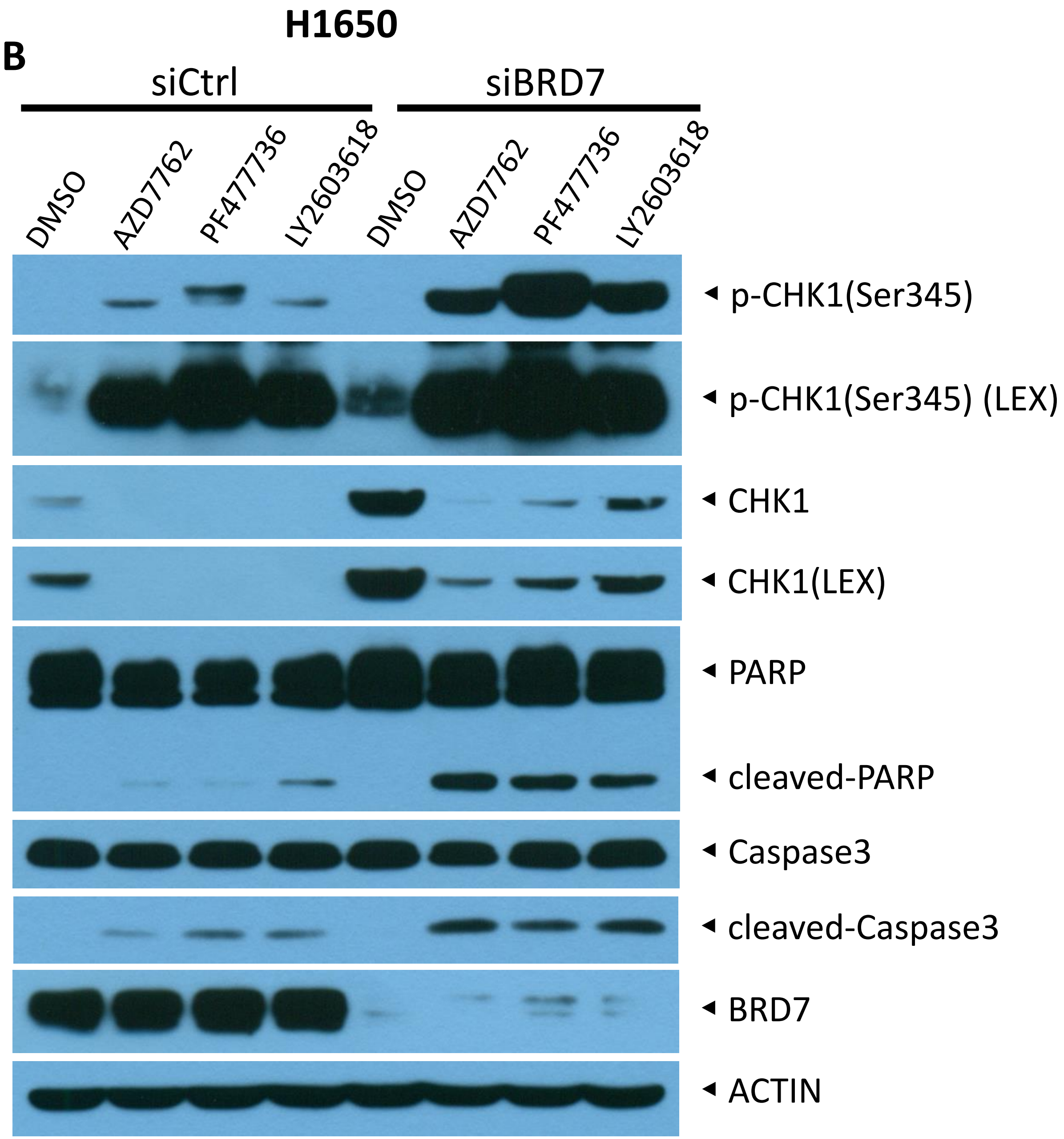

Figure 5C

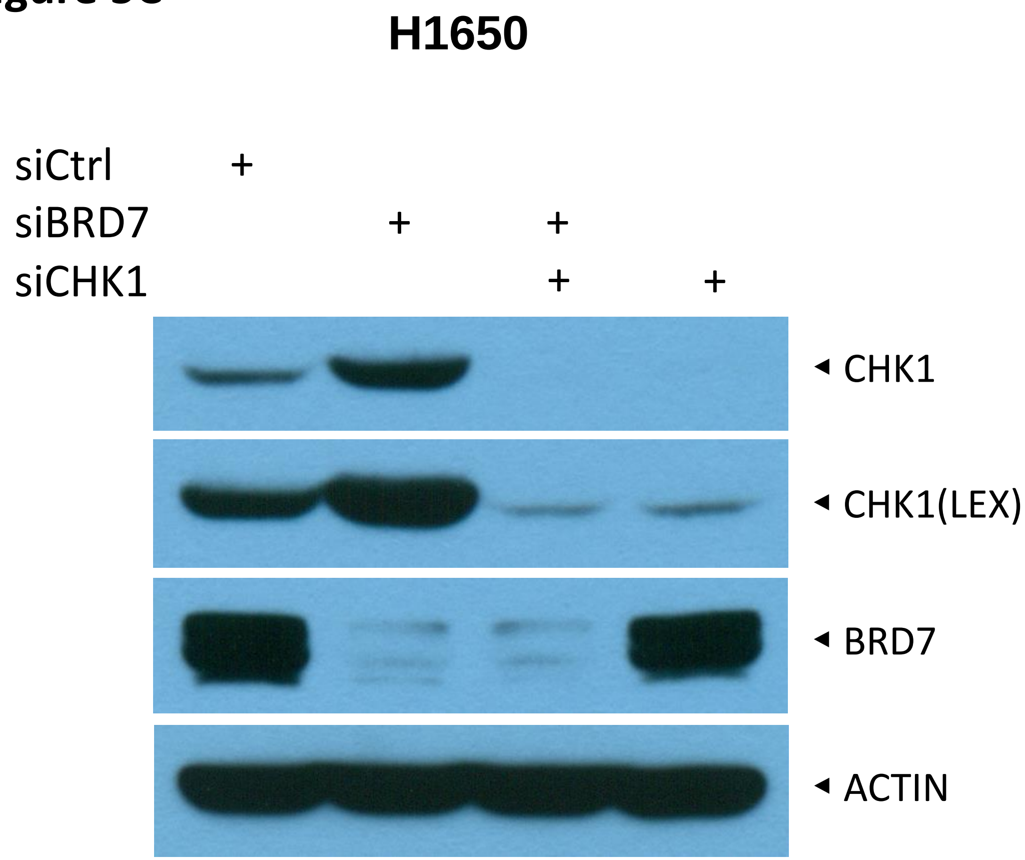

Figure 6A

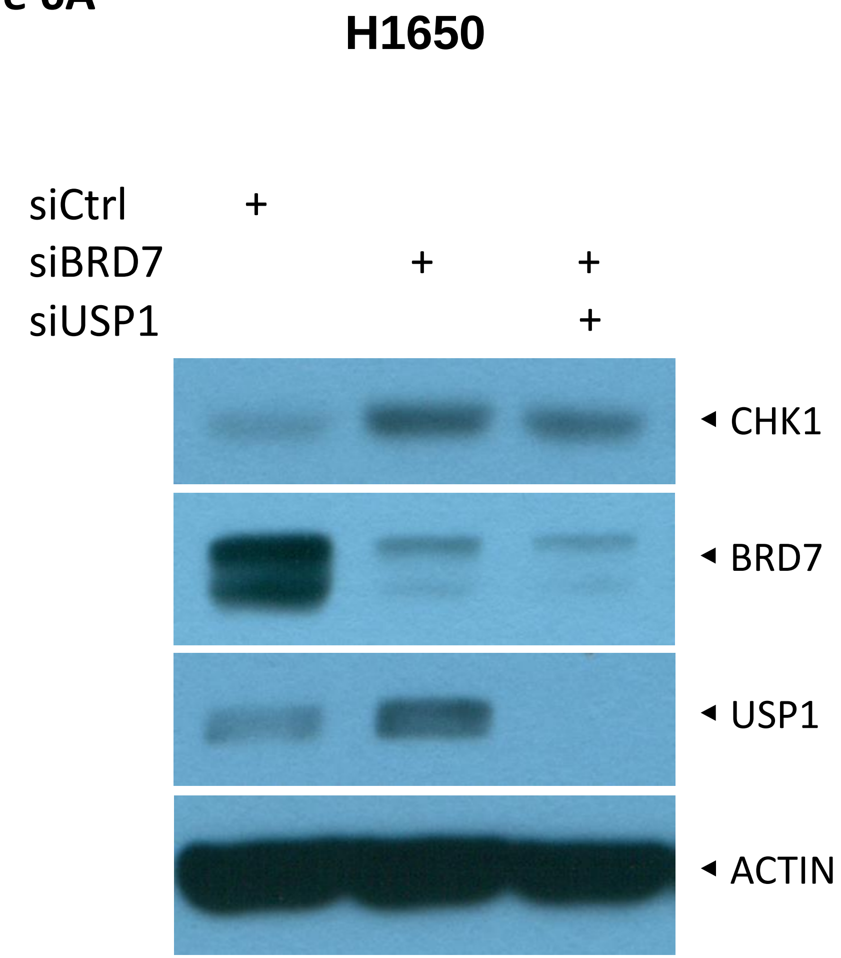

Figure S1A

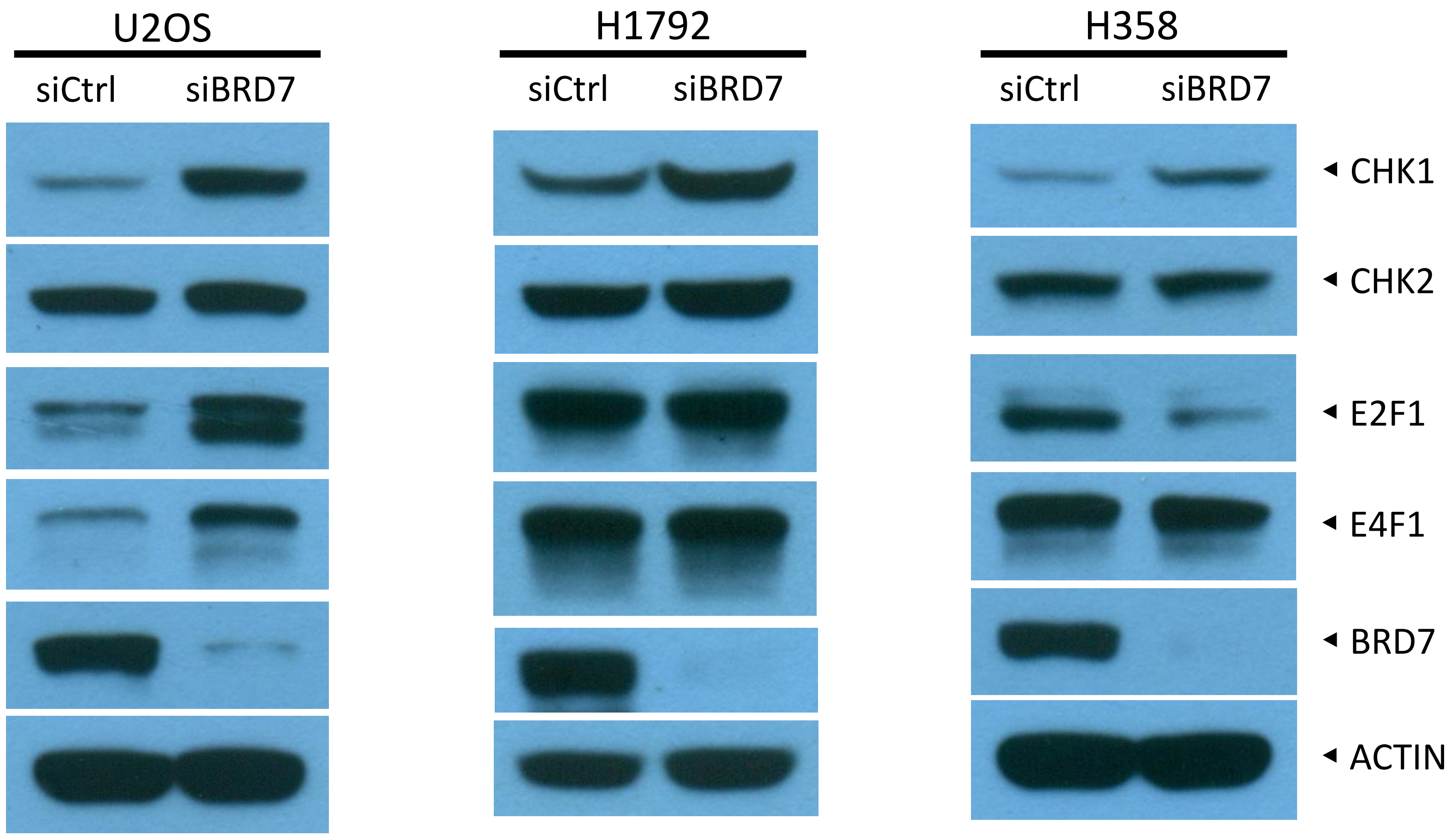
U2OS
siCtrl
siBRD7
H1792
siCtrl
siBRD7
H358
siCtrl
siBRD7
CHK1
CHK2
E2F1
E4F1
BRD7
ACTIN

Figure S1A

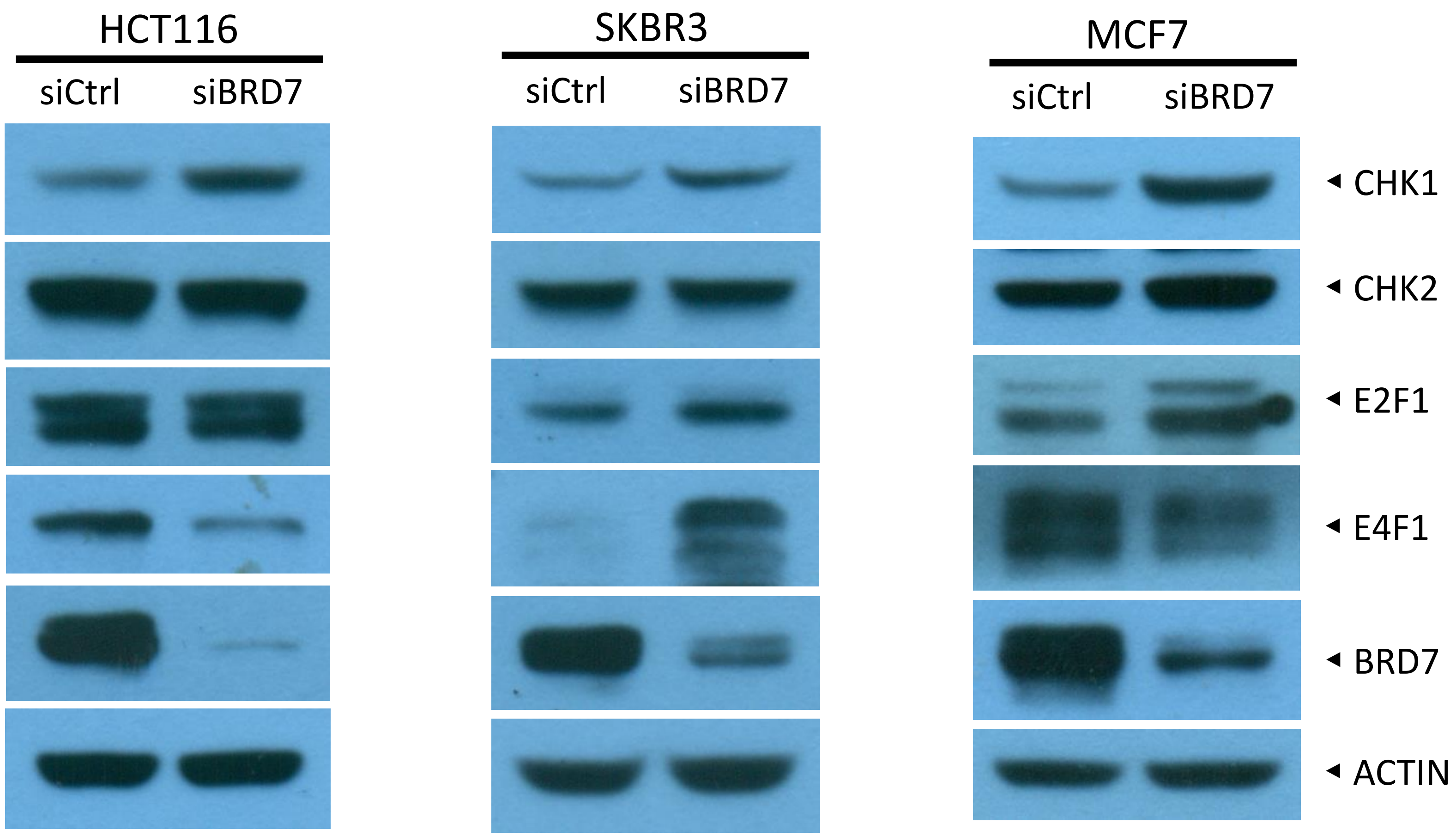

HCT116
siCtrl
siBRD7
SKBR3
siCtrl
siBRD7
MCF7
siCtrl
siBRD7
CHK1
CHK2
E2F1
E4F1
BRD7
ACTIN

Figure S1B

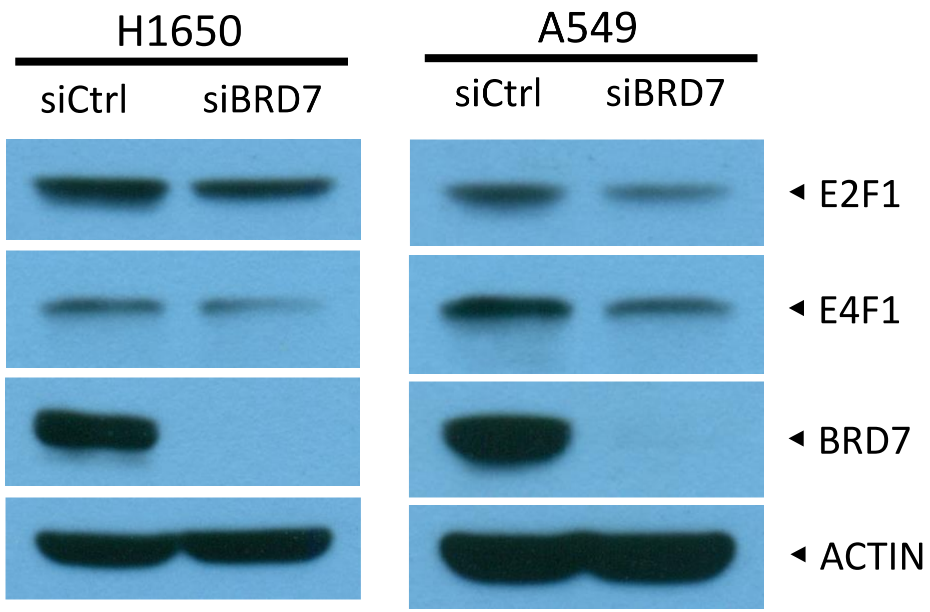
H1650
siCtrl
siBRD7
A549
siCtrl
siBRD7
E2F1
E4F1
BRD7
ACTIN

Figure S1C

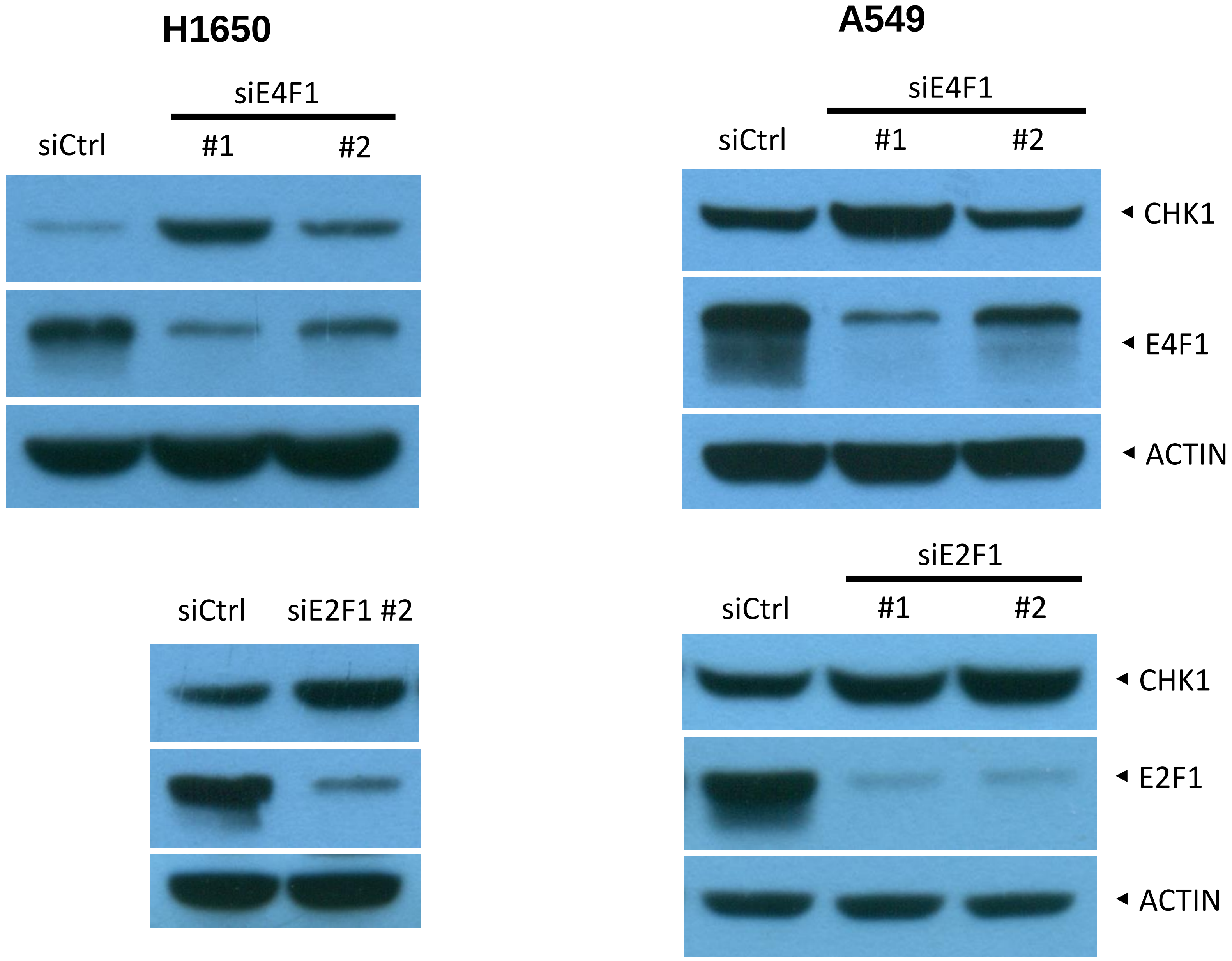

H1650
siE4F1
siCtrl
#1
#2
siCtrl
siE2F1 #2
A549
siE4F1
siCtrl
#1
#2
CHK1
E4F1
ACTIN
siE2F1
siCtrl
#1
#2
CHK1
E2F1
ACTIN

Figure S2

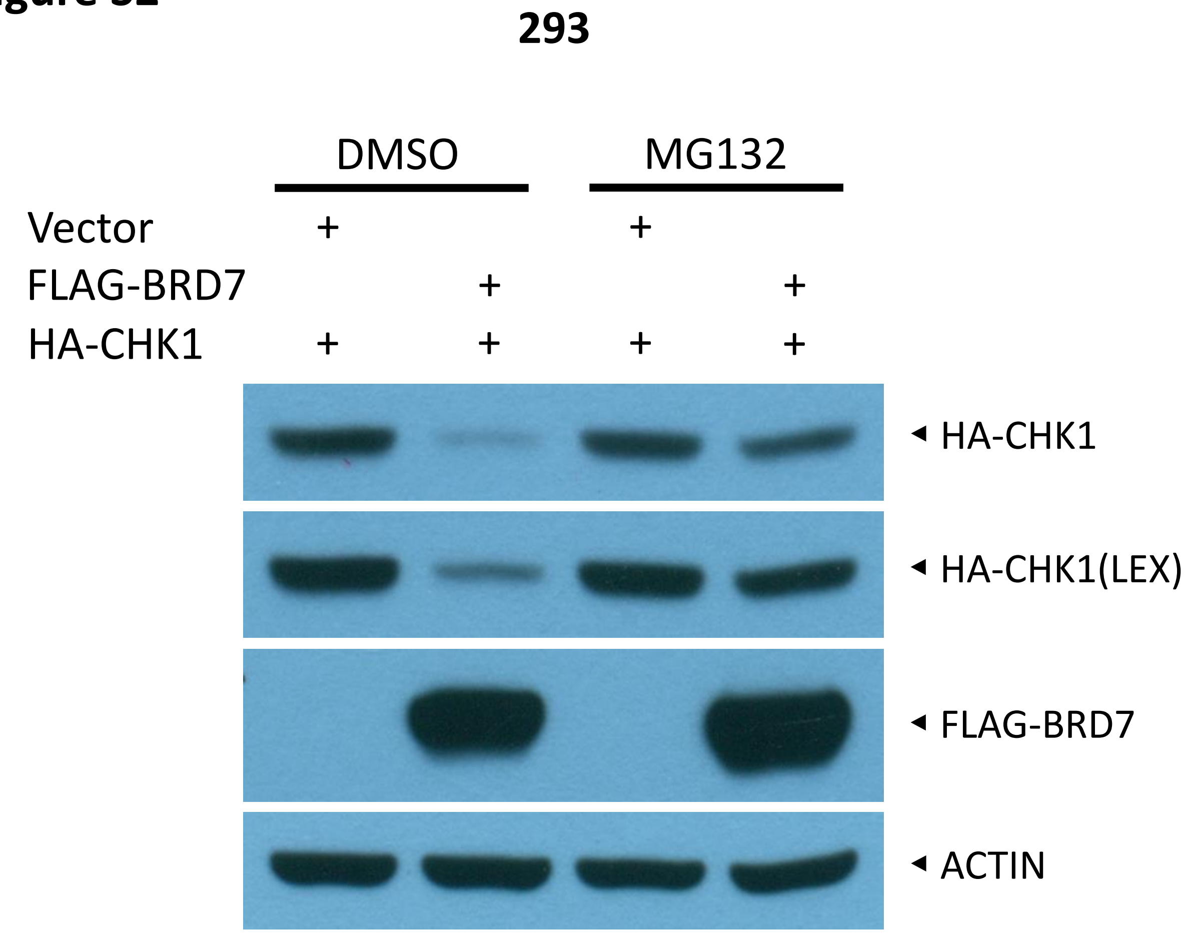
293
DMSO
MG132
Vector
FLAG-BRD7
HA-CHK1
HA-CHK1
HA-CHK1(LEX)
FLAG-BRD7
ACTIN

Figure S3A

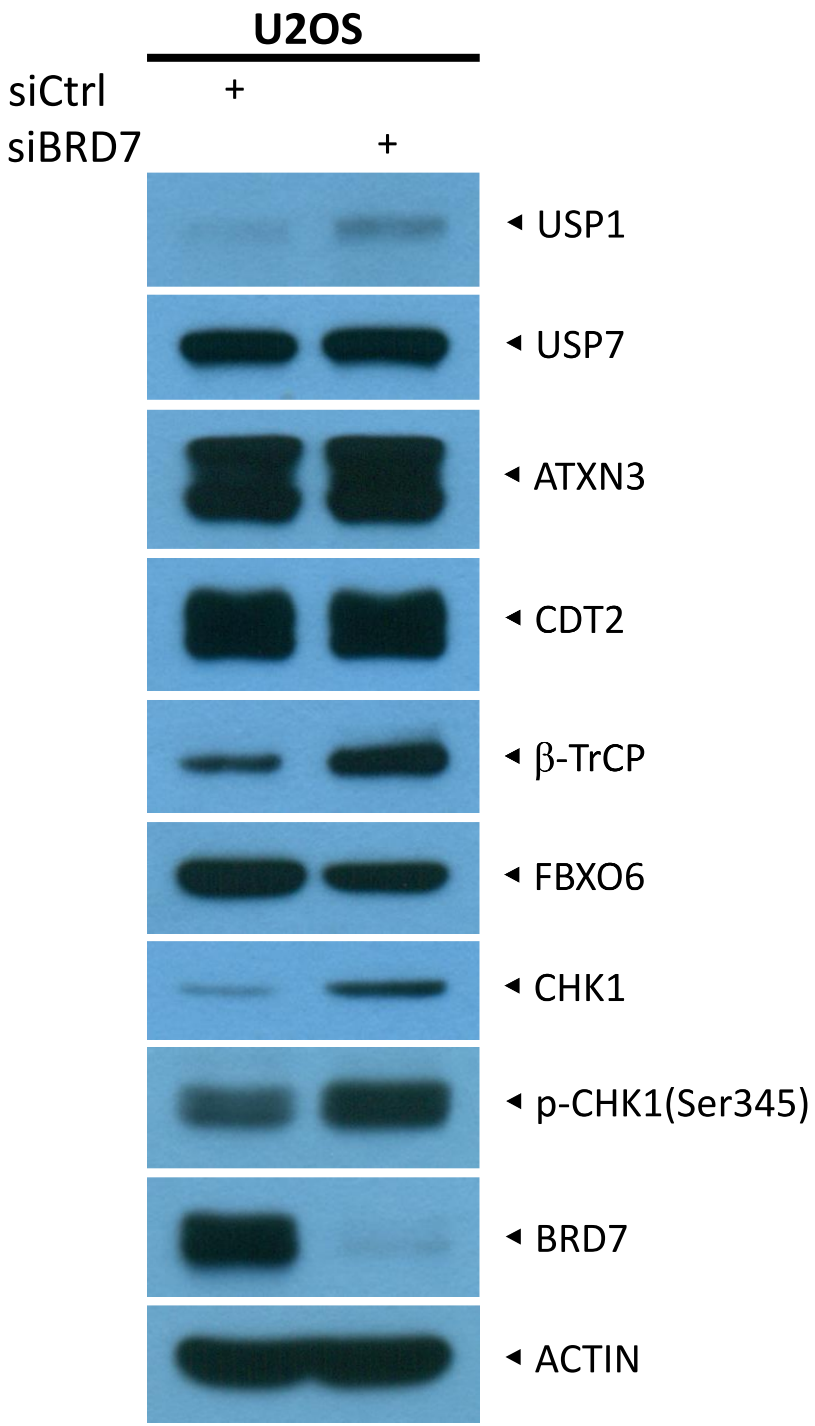

Figure S3B

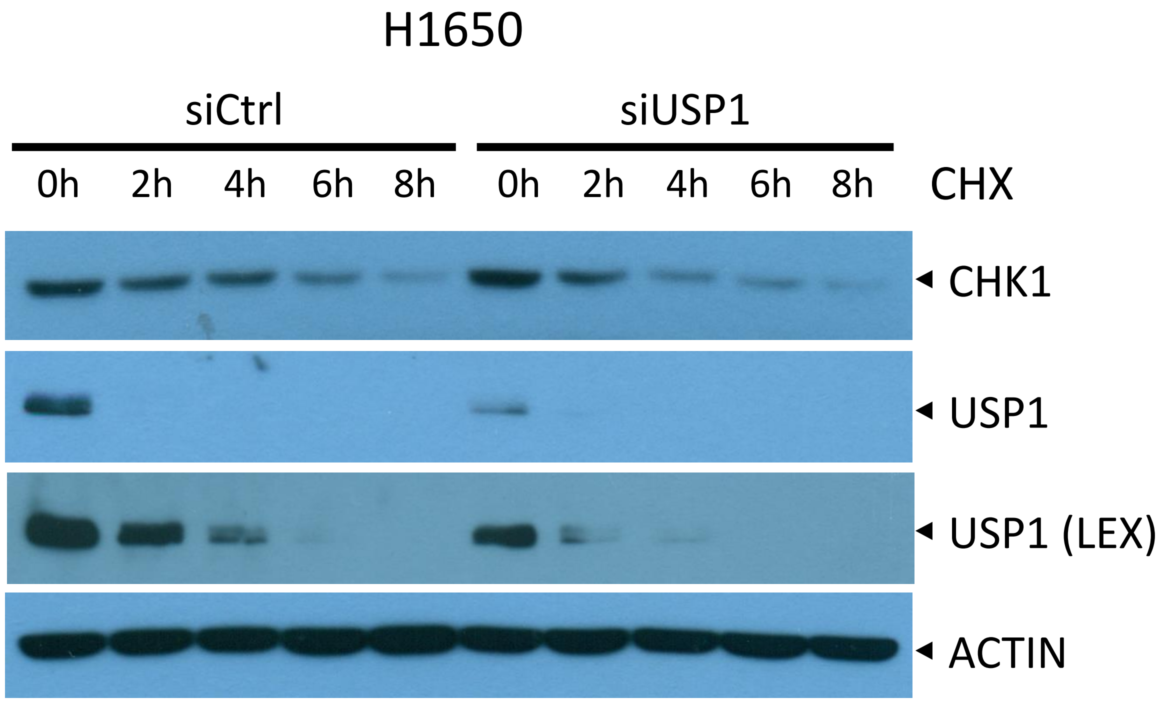

Figure S3C

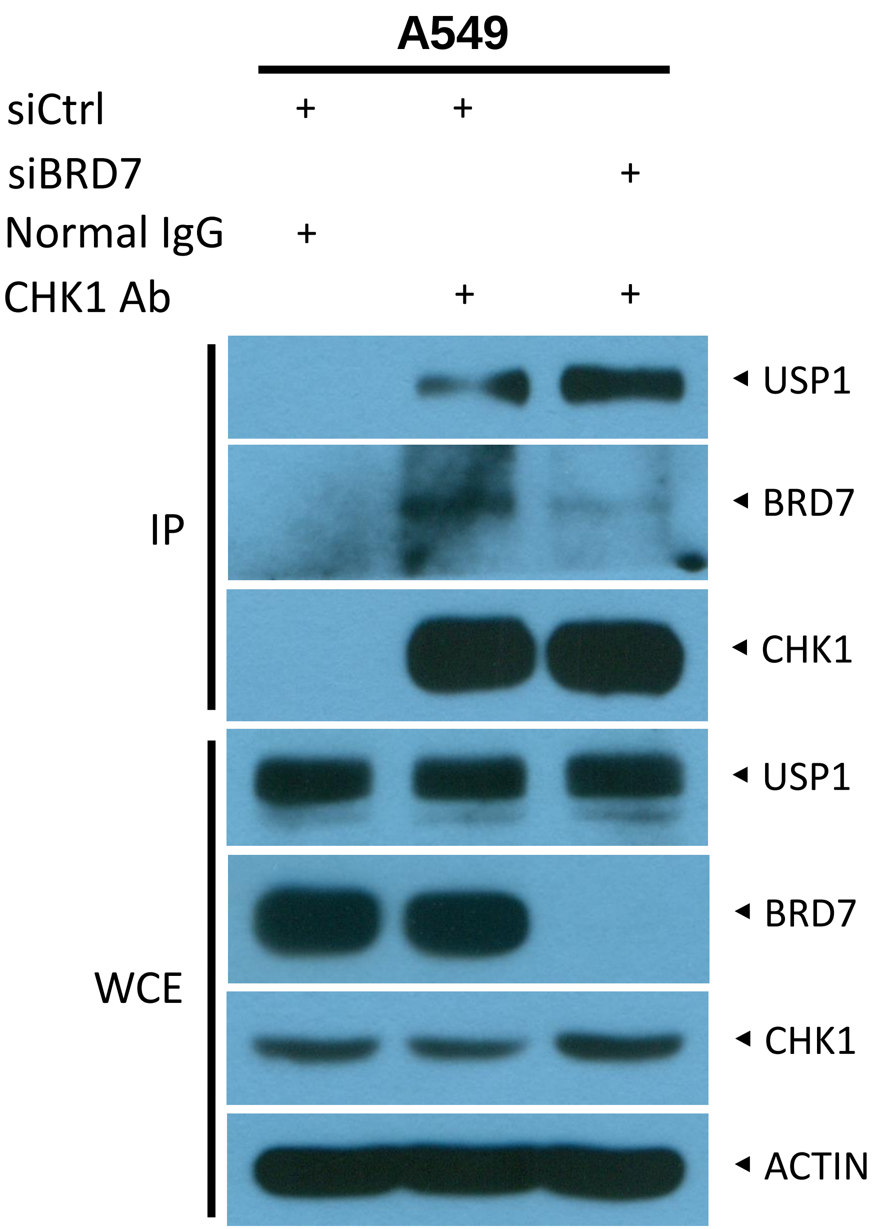
A549
siCtrl
+
+
siBRD7
+
Normal IgG
+
CHK1 Ab
+
+
IP
WCE
USP1
BRD7
CHK1
USP1
BRD7
CHK1
ACTIN

Figure S4A

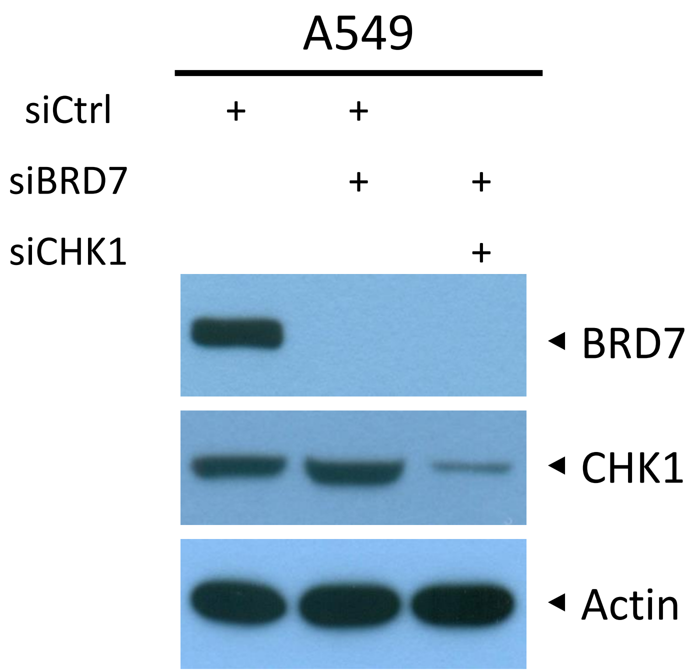

Figure S5C

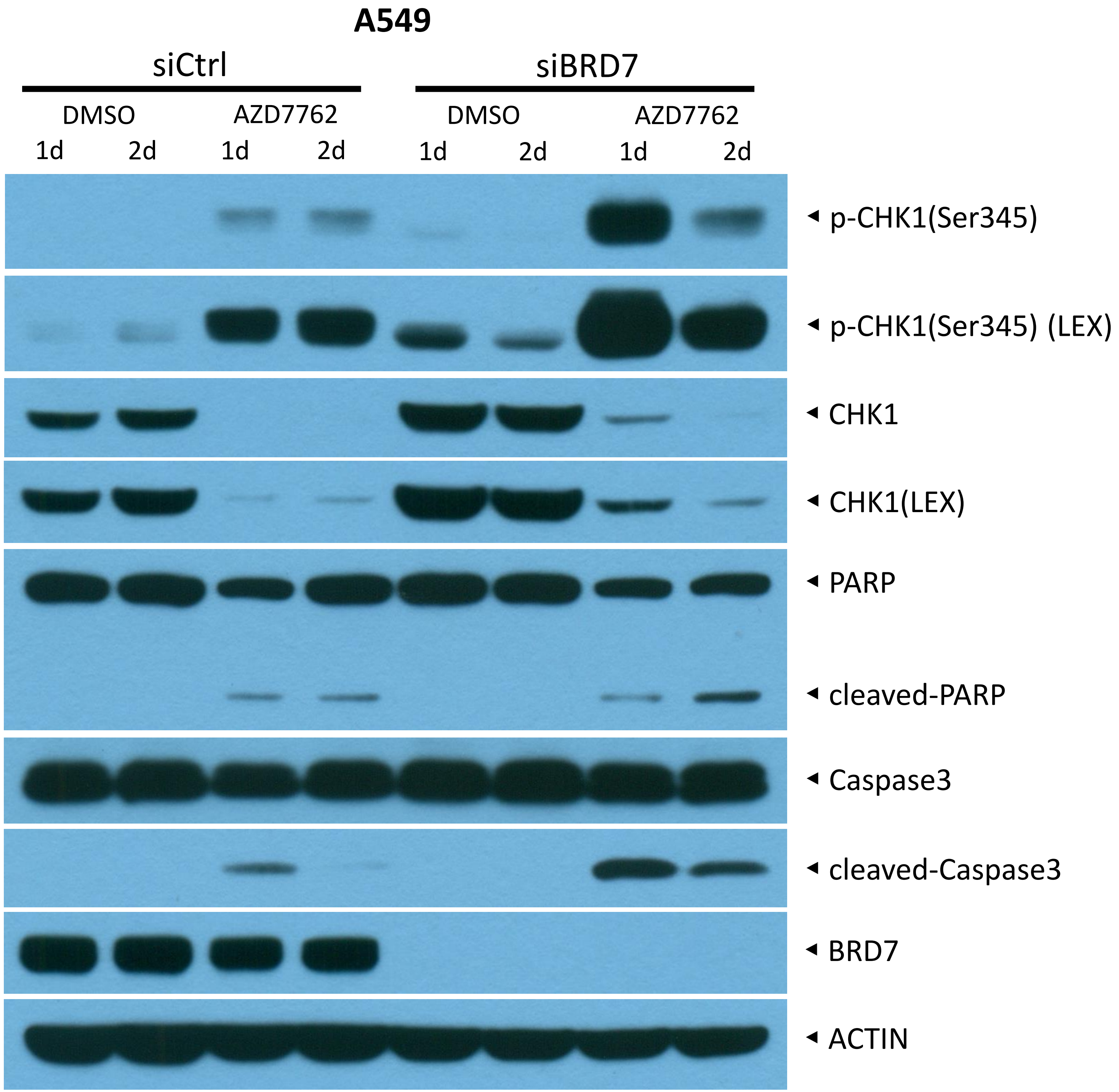
A549
siCtrl
siBRD7
DMSO
AZD7762
DMSO
AZD7762
1d
2d
1d
2d
1d
2d
1d
2d
p-CHK1(Ser345)
p-CHK1(Ser345) (LEX)
CHK1
CHK1(LEX)
PARP
cleaved-PARP
Caspase3
cleaved-Caspase3
BRD7
ACTIN

Figure S5C

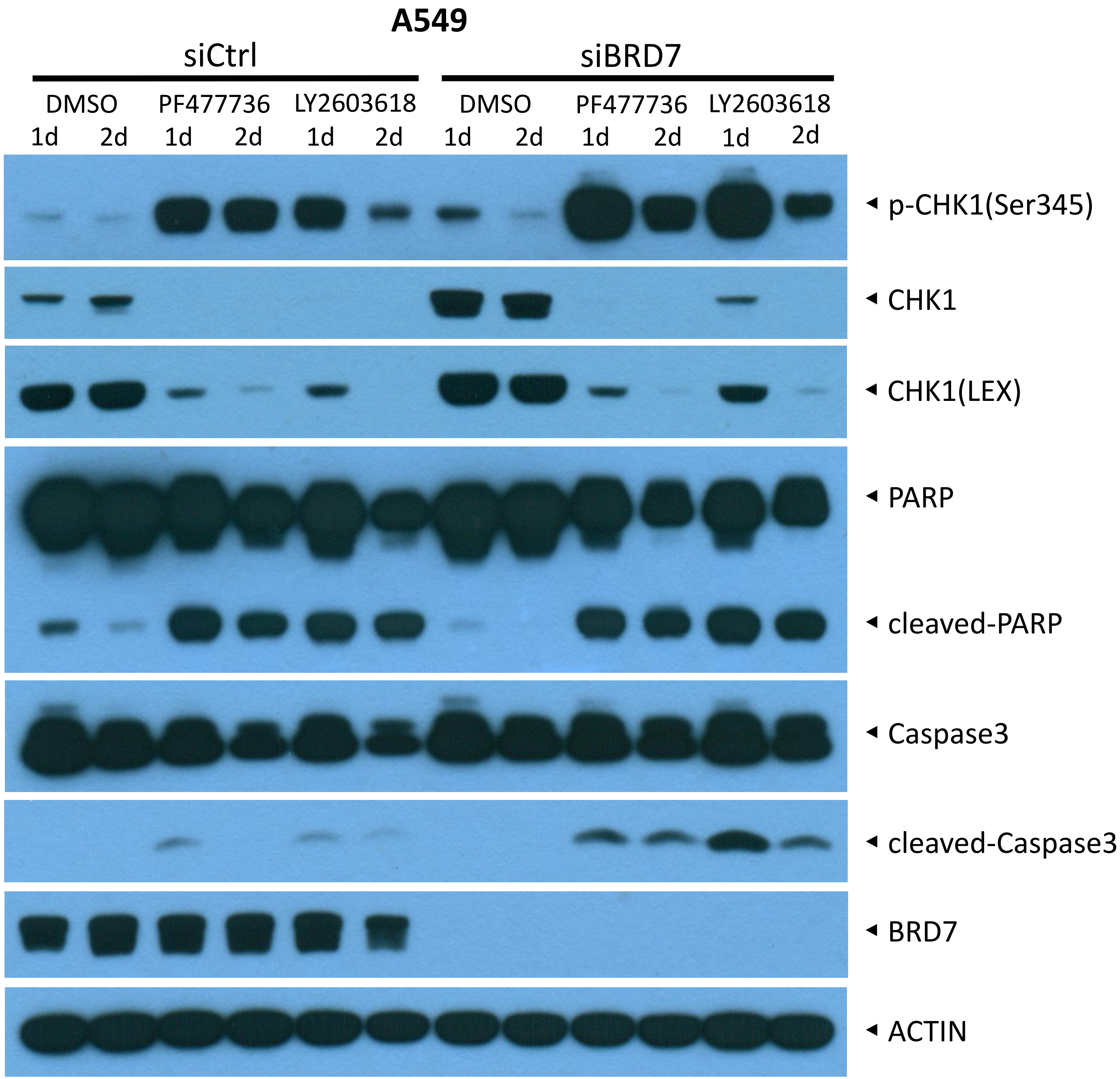
A549
siCtrl
siBRD7
DMSO
PF477736
LY2603618
DMSO
PF477736
LY2603618
1d 2d 1d 2d 1d 2d 1d 2d 1d 2d 1d 2d
p-CHK1(Ser345)
CHK1
CHK1(LEX)
PARP
cleaved-PARP
Caspase3
cleaved-Caspase3
BRD7
ACTIN
