# Supplementary material for: BRD7 suppresses tumor chemosensitivity to CHK1 inhibitors by inhibiting USP1-mediated deubiquitination of CHK1
Source: Cell Death Discov. 2023 Aug 25;9:313. doi: 10.1038/s41420-023-01611-x (PMC10457387; doi:10.1038/s41420-023-01611-x)
Supplement: Supplementary file 9 — Author Contribution Statement [file 41420_2023_1611_MOESM9_ESM.pdf]

DECLARATION OF CONTRIBUTIONS TO ARTICLE

ADMC

Manuscript Number: CDDISCOVERY-23-0338R

Journal Name: *Cell Death Discovery* (the 'Journal')

Proposed Title of the Contribution:

BRD7 suppresses tumor chemosensitivity to CHK1 inhibitors by inhibiting USP1-mediated deubiquitination of CHK1 (the 'Contribution)

Author(s):

Lemin Li, Linchen Wang, Dian Liu, Yongchao Zhao (the 'Authors')

For all *CDDis*covery articles, each person named as an author in the published version must be able to show he or she has contributed substantially to the article.

Authorship credit should be based on 1) substantial contributions to conception and design, acquisition of data, or analysis and interpretation of data; 2) drafting the article or revising it critically for important intellectual content; and 3) final approval of the version to be published. Authors should meet conditions 1, 2 and 3.

Any person who cannot be shown to have made a substantial contribution to the article cannot be listed as an author in the final version. The name of any person who is deemed to have made a minor contribution can, however, appear in the Acknowledgments section of the article.

Please complete the table below to indicate the contributions of all named authors to the manuscript.

Author Full Name:	Specification of Contribution to the Manuscript:
Lemin Li	acquisition and analysis of the data, drafting of the manuscript
Linchen Wang	acquisition and analysis of the data, drafting of the manuscript
Dian Liu	acquisition of the data
Yongchao Zhao	conception and design, analysis and interpretation of the data, drafting and revision of the manuscript

Please complete the table below to indicate the contributions of all named authors to the figures.

Figure 1:
Lemin Li, Yongchao Zhao

Figure 2:
Lemin Li, Yongchao Zhao

Figure 3:
Lemin Li, Linchen Wang, Dian Liu, Yongchao Zhao

Figure 4:
Linchen Wang, Yongchao Zhao

Figure 5:
Lemin Li, Yongchao Zhao

Figure 6:
Lemin Li, Yongchao Zhao

Signed for and on behalf of the Author(s):	Print Name:	Date:
Yongchao Zhao	Yongchao ZHAO	08/10/2023
